# Inhaled nitric oxide in preterm infants with respiratory disease: a systematic review and meta-analysis

**DOI:** 10.1186/s40001-025-03008-1

**Published:** 2025-08-29

**Authors:** Kai Zhou, Weipeng Xu, Danrui Li, CheokUn Lao, Shiqian Zou, Shixian Liu, Bingxiao Li, Fangfang Zeng, Sui Zhu, Shasha Han

**Affiliations:** 1 Department of Neonatology and Pediatrics, The First Affiliated Hospital of Jinan University, 613 W. Huangpu Avenue, Guangzhou, 510630 Guangdong China; 2 Department of Public Health and Preventive Medicine, School of Medicine, Jinan University, Guangzhou, Guangdong China; 3 Faculty of Medicine, International School, Jinan University, Guangzhou, Guangdong China; 4 State Key Laboratory of Ophthalmology, Zhongshan Ophthalmic Center, Sun Yat-Sen University, Guangzhou, Guangdong China

**Keywords:** Inhaled nitric oxide, Preterm infants, Bronchopulmonary dysplasia, Death, Meta-analysis

## Abstract

**Background:**

Inhaled nitric oxide (iNO) has been shown to be effective in term and near-term infants with specific respiratory diseases. The effects and potential risks of iNO differ substantially in preterm infants with special pathophysiology. Specific study in this population is necessary.

**Purpose:**

To assess the short-term and long-term effects of iNO in preterm infants with respiratory diseases such as respiratory failure and respiratory distress syndrome.

**Methods:**

We conducted a meta-analysis of randomized controlled trials and cohort studies comparing iNO with placebo or blank control in preterm infants with respiratory diseases.Databases including PubMed, the Cochrane Library, Scopus, and Web of Science were searched from their inception until May 2025. The primary outcomes were death before discharge, death at 36 weeks’ postmenstrual age (PMA), bronchopulmonary dysplasia (BPD) and death or BPD.

**Results:**

Thirty-one trials met the inclusion criteria. iNO reduced the incidence of death or BPD (risk ratio [RR] 0.94, 95% confidence interval [CI] 0.88 to 0.99, 6 studies, 1954 infants) and BPD in the RCT subgroup (RR 0.91, 95% CI 0.84 to 0.99, 8 studies, 2196 infants).Increases of oxygenation index (Standardized mean difference (SMD)  − 0.62, 95% CI − 0.81 to − 0.43, 2 studies, 441 infants) and Partial pressure of oxygen (PaO_2_) (SMD 0.68, 95% CI 0.49 to 0.87, 2 studies, 441 infants) of preterm infants at 5 ppm initial concentration were observed in the iNO group. The iNO group demonstrated a higher risk of retinopathy of prematurity (RR 1.08, 95% CI 1.01 to 1.16, 17 studies, 7220 infants). No significant differences were observed in other neonatal morbidities or adverse events.

**Conclusions:**

iNO was associated with a reduced risk of death or BPD and a probably reduction of BPD. There was no effect on other morbidities or adverse events. Data on long-term respiratory and neurodevelopment outcomes are critically needed to further evaluate NO's efficacy in preterm infants, particularly Extremely low birth weight infants.

**Supplementary Information:**

The online version contains supplementary material available at 10.1186/s40001-025-03008-1.

## Introduction

In early neonates, respiratory distress is very common with an incidence rate of up to 7% of newborn infants, resulting in significant numbers of infants admitted to neonatal intensive care units [1]. Maintaining adequate oxygenation is paramount in managing these infants, as hypoxemia exacerbates pulmonary vasoconstriction and respiratory acidosis, creating a vicious cycle of clinical deterioration. This critical need for oxygenation stems from the pathophysiology of key respiratory diseases, including early postnatal respiratory distress syndrome (RDS), meconium aspiration syndrome (MAS), hypoxic respiratory failure (HRF), and persistent pulmonary hypertension of the newborn (PPHN). In addition to mechanical ventilation to ensure lung ventilation, targeted interventions to optimize pulmonary blood flow—such as inhaled nitric oxide (iNO)—are essential. Nitric oxide (NO), produced by vascular endothelial cells, dilates pulmonary vessels, improves ventilation-perfusion matching, and exerts anti-inflammatory effects, thereby addressing both the hemodynamic and inflammatory components of respiratory diseases [2]. iNO has been consistently shown to reduce mortality or the need for extracorporeal membrane oxygenation (ECMO) in term and late-preterm infants [3]. The United States Food and Drug Administration has approved iNO for the treatment of PPHN in infants > 34 weeks gestational age, confirming its safety in term and near-term infants [4]. While iNO is well-established for term infants, its role in preterm neonates remains controversial due to distinct pathophysiology. Currently, administration of iNO to infants ≤ 34 weeks’ gestation is off-label and primarily limited to emergency rescue situations. In 2011, the National Institutes of Health (NIH) issued a consensus statement discouraging the routine use of iNO in preterm infants due to equivocal evidence on pulmonary outcomes, survival, and long-term neurodevelopment [5]. A survey across Australia and New Zealand revealed that nearly all neonatal units employed iNO as early rescue therapy for preterm infants with HRF [6]. Similarly, a Japanese questionnaire study reported that iNO was predominantly administered to extremely preterm infants as early rescue treatment for PPHN [7]. There is much evidence support the use of iNO in premature infants ≤ 34 weeks gestation [8–12], but high-quality evidence remains insufficient. Given the significant differences in pulmonary development, further investigation of iNO therapy for preterm infants is both crucial and urgently needed [13].

Previous meta-analyses have not fully addressed the long-term effects of iNO on preterm infants, nor have they fully explored the effects of iNO on different disease subgroups of preterm infants, and have not included different types of studies. This updated meta-analysis aims to fill these gaps by including long-term results, conducting detailed subgroup analyses, incorporating studies of different diseases, and including both RCTs and observational studies, thereby gaining a more detailed understanding of the therapeutic effect of iNO across preterm infants.

## Methods

### Search strategy and information resources

We searched PubMed, Scopus, Web of Science, and Cochrane Library from the inception of publication to May 2025 using the MeSH terms “Infant, Premature” combined with keywords “Nitric Oxide” and limited to English language articles. We combined the population free words and intervention free words by OR, and combined the population and intervention query by AND. We also added articles from the included articles of previous meta-analyses that met our eligibility criteria. The specific search queries are detailed in Online Resource 1 “Databases search queries”.

### Inclusion criteria

1. Randomized Controlled trials (RCTs) and cohort studies that included preterm infants with respiratory diseases (RDS, pulmonary hypertension, pulmonary hypoplasia, respiratory failure).
2. Studies where the intervention group treated with iNO and the control group did not use iNO.

### Exclusion criteria

1. Studies where the control group received iNO therapy.
2. Studies where the control group used other drugs different from the intervention group or did not receive a placebo alone.
3. Studies with mixed gestational ages (e.g., term and preterm infants) that lacked stratified outcome data for preterm subgroups.
4. Studies without a clear iNO therapy protocol.
5. Studies with incomplete data.

### Data extraction and analysis

Data extraction followed PRISMA 2020 recommendations, with two authors independently performing each step to ensure methodological rigor. The completed PRISMA checklist was provided in Online Resource 2. Two authors (Kai Zhou and Weipeng Xu) independently screened titles and abstracts. Studies identified as potentially relevant were then retrieved for full-text review. The same two authors independently assessed the full text of these articles against the inclusion and exclusion criteria. Disagreements were resolved by discussion, and if necessary, a third author (Shasha Han) was consulted to reach a consensus. Extracted data included the study and population characteristics, intervention and comparison protocols, outcome measures, and measurement tools with corresponding results.

The risk of bias of all the RCT trials was assessed based on the Cochrane Collaboration tools (RoB 1.0). We assessed the methodological quality of each trial based on 7 criteria: random sequence generation, allocation concealment, blinding of participants and personnel, blinding of outcome assessment, incomplete outcome data, and other bias. For criteria defined as ‘high’, ‘low’, or ‘unclear’, ‘high’ indicates clear flaws that may compromise results, ‘low’ signifies methods aligned with best practices to ensure reliability, and ‘unclear’ reflects insufficient information to determine risk [14]. All observational studies were evaluated using the Newcastle–Ottawa quality assessment scale. The assessment focused on three critical domains: (1) population selection, (2) comparability between exposed and comparator groups, and (3) outcome assessment (for cohort studies) or exposure assessment (for case–control studies). Each domain was rated as poor, fair, or good, with studies receiving an overall risk-of-bias classification of high, moderate, or low. One study received 9 stars at most and zero star at least. Studies obtaining five or more stars were considered of good quality [15]. The certainty of evidence was evaluated using the Grading of Recommendations Assessment, Development, and Evaluation (GRADE) framework (https://www.gradepro.org), which provides a transparent and systematic methodology to rate evidence quality from high to very low by assessing five domains: risk of bias (methodological limitations in included studies), inconsistency (heterogeneity in results across studies), indirectness (applicability to the research question), imprecision (wide confidence intervals or insufficient sample size), and publication bias (potential missing evidence due to selective reporting), ultimately categorizing the evidence into four levels of certainty—high (further research is very unlikely to change confidence in the effect estimate), moderate (further research is likely to significantly alter the effect estimate), low (the true effect is likely substantially different from the estimated effect), and very low (significant uncertainty exists in the effect estimate).

### Outcome measures

#### Primary outcomes

Death before discharge, death at 36 weeks’ postmenstrual age (PMA), bronchopulmonary dysplasia (BPD, defined as use of supplemental oxygen treatment or oxygen plus respiratory support at 36 weeks PMA [16]), and death or BPD.

#### Secondary outcomes

Improvement in OI and PaO_2_ 30 min after the use of iNO, visual impairment outcomes (including ROP and visual impairment), respiratory system outcomes (including oxygen use and complications), nervous system outcomes (including long-term and short-term outcomes), NEC, symptomatic PDA and vasopressor use, postnatal steroids, duration of hospitalization in survivors.

### Statistical analysis

In this meta-analysis, effect estimates for dichotomous outcomes were expressed as risk ratios (RR) with 95% confidence intervals (CI), while standardized mean differences (SMD) with 95% CI were calculated for continuous outcomes. Heterogeneity across studies was quantified using the *I*^*2*^ statistic, with *I*^*2*^ ≤ 50% indicating low heterogeneity and justifying a fixed-effects model, whereas *I*^*2*^ > 50% prompted the use of a random-effects model. Statistical significance was set at *p* < 0.05. To address potential missing data, sensitivity analyses were conducted by iteratively excluding individual studies to assess their influence on pooled estimates. Additional subgroup analyses were performed to explore heterogeneity sources, including study design (RCTs vs non-RCTs), target populations (different respiratory diseases), and dosage of iNO to assess whether the study design, the types of diseases and the dosage of iNO influenced the observed effects. Publication bias was evaluated through funnel plot, complemented by Begg’s rank correlation and Egger’s regression tests. All analyses were implemented in R software (version 4.2.3).

## Results

### Study characteristics

**Fig. 1:**
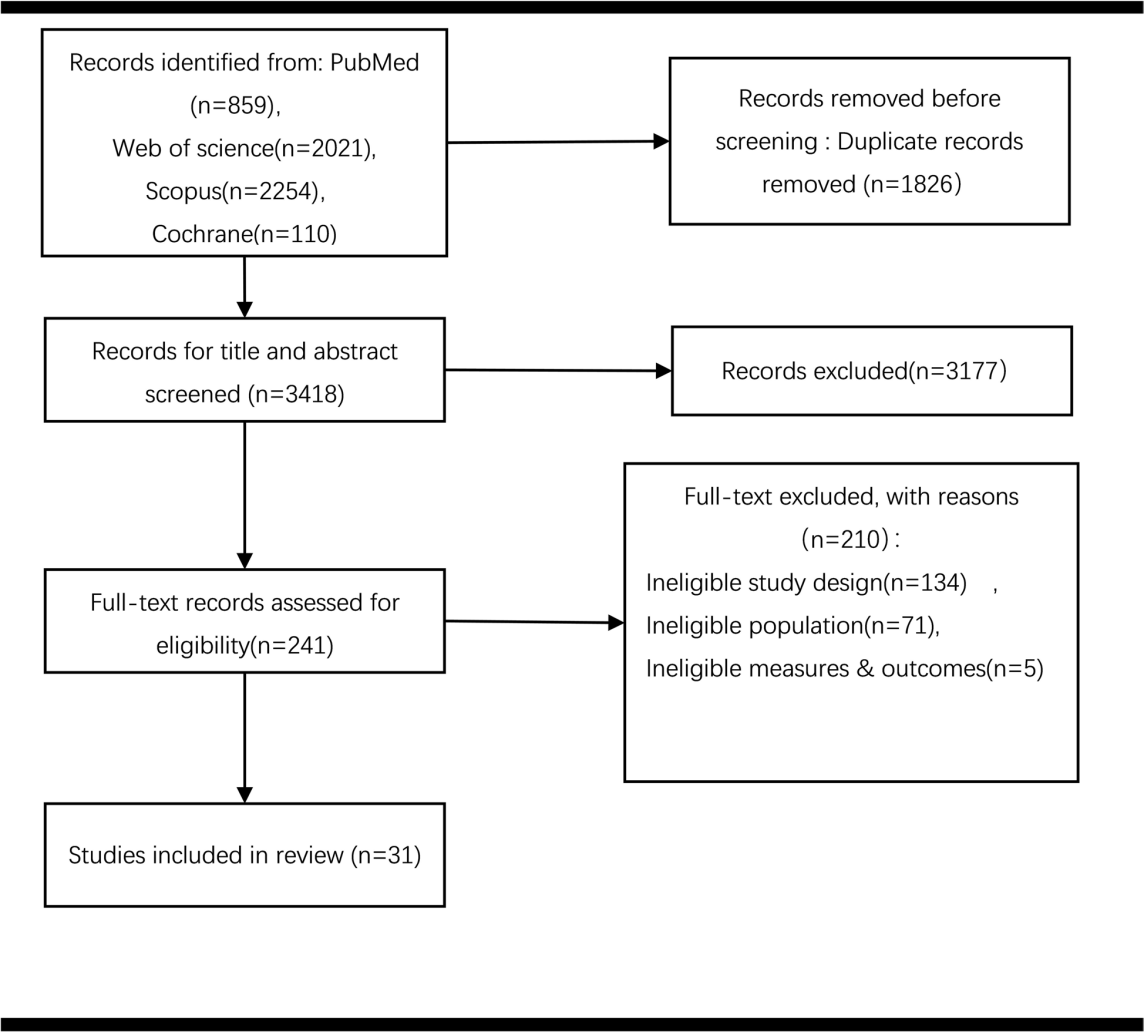
PRISMA flow diagram of included and excluded studies

In this updated meta-analysis, we retrieved a total of 5,244 citations, including 859 articles from PubMed, 110 from Cochrane, 2,254 from Scopus, and 2,021 from Web of Science. After importing all records into EndNote and removing duplicates, 3,418 papers remained for screening. Ultimately, we included 20 RCTs and 11 cohort studies, comprising 20,080 preterm infants (mostly ≤ 34 weeks gestation), which compared the use of iNO against placebo or blank control. The selection process is illustrated in Fig. 1. Among them, short-term outcomes were reported in 20 studies, while 5 studies reported long-term outcomes, and 6 studies reported both. The enrolled preterm infants exhibited several specific diseases, such as respiratory failure (RF), RDS, pulmonary hypoplasia (PH), PPHN, or a combination of these. After reviewing and extracting the details, we summarized the characteristics of each included study in Table 1.

**Table 1:** Characteristic of the studies

Author	Year	Design	Outcome type	Country	GA (weeks; T/C)	BW (T/C)	n (T/C)	Specific disease	Control	iNO dosage (start)	iNO dosage (max)
Kinsella	1999	RCT	Short-term	American	27.1 ± 2.5; 26.8 ± 2.5	1040 ± 461 g; 988 ± 387 g	80 (48/32)	Respiratory failure	Blank	5	-
Schreiber	2003	RCT	Short-term	American	27.4 ± 2.5; 27.0 ± 2.8	1017 ± 369 g; 949 ± 387 g	207 (105/102)	RDS	Placebo	10	10
Uga	2004	Cohort	Short-term	Japan	27.2 ± 2.2; 25.8 ± 2.4	996 ± 294 g; 809 ± 316 g	18 (8/10)	Pulmonary hypoplasia	Blank	-	40
Hamon	2005	RCT	Short-term	France	27.3 ± 0.4; 27.9 ± 0.4	1083 ± 058 g; 1102 ± 054 g	76 (37/39)	Respiratory failure	Placebo (nitrogen)	5	10
Meurs	2005	RCT	Short-term	American	26 ± 2; 26 ± 2	840 ± 264 g; 837 ± 260 g	420 (210/210)	Respiratory failure	Placebo	5	10
Elbourne	2005	RCT	Short-term and long-term	UK	27.4 ± 2.6, 26.3 ± 2.4	1066 ± 395, 890 ± 343 g	108 (55/53)	Respiratory failure	Blank	5	40
Hascoet	2005	RCT	Short-term	France and Belgium	–	–	860 (415/445)	Respiratory failure	Placebo (nitrogen)	5	10
Mestan	2005	RCT	Long-term	American	27.5 ± 2.4; 27.2 ± 2.6	1026 ± 366 g; 958 ± 356 g	138 (70/68)	RDS	Placebo	10	10
Kinsella	2006	RCT	Short-term	American	25.6 ± 1.7; 25.6 ± 1.8	796 ± 190 g; 788 ± 185 g	793 (393/395)	Respiratory failure	Placebo (nitrogen)	5	-
Ballard	2006	RCT	Short-term	American	26 ± 1.5; 26 ± 1.5	766 ± 161 g; 759 ± 155 g	582 (294/288)	Premature	Placebo	20	20
Dani	2006	RCT	Short-term	Italy	26.3 ± 2.6; 26.7 ± 1.9	937.0 ± 298.0 g; 825.0 ± 299.3 g	40 (20/20)	RDS	Placebo	10	10
Meurs	2007	RCT	Short-term and long-term	American	31.1 ± 1.2; 31.4 ± 1.1	1970 ± 391 g; 2168 ± 441 g	39 (14/15)	Respiratory failure	Placebo	5	10
Hintz	2007	RCT	Long-term	American	25.9 ± 2.3; 25.9 ± 2.2	835 ± 265 g; 830 ± 261 g	420 (210/210)	Respiratory failure	Placebo	5	10
Su	2008	RCT	Short-term	China Taiwan	27.4 ± 2.3; 27.9 ± 1.8	1020 ± 230 g; 1050 ± 210 g	65 (32/33)	Respiratory failure	Placebo	5	20
Hibbs	2008	RCT	Long-term	American	25.8 ± 1.4; 25.7 ± 1.5	763 ± 163 g; 762 ± 150 g	455 (230/225)	Premature	Placebo	20	20
Valerie	2009	Cohort	Short-term	American	27 ± 2; 29 ± 3	1039 ± 355 g; 1179 ± 369 g	12 (6/6)	Pulmonary hypoplasia	Placebo	5	10
Mercier	2010	RCT	Short-term	European Union	26.4 ± 1.3; 26.6 ± 1.3	851 ± 207 g; 864 ± 192 g	800 (399/401)		Placebo (nitrogen)	5	-
Walsh	2010	RCT	Long-term	American	25.8 ± 1.4; 25.7 ± 1.5	765 ± 163 g; 764 ± 153 g	477 (243/234)	Premature	Placebo	20	20
Kinsella	2014	RCT	Short-term	American	27.5 ± 1.6; 27.3 ± 1.8	961 ± 186 g; 968 ± 159 g	124 (59/65)	Premature	Placebo (nitrogen)	10	10
Durrmeyer	2015	RCT	Long-term	European Union	26.5 ± 1.3; 26.6 ± 1.3	858.0 ± 211 g; 883.9 ± 188.3 g	792 (395/397)	Respiratory failure	Placebo (nitrogen)	5	-
Jiang	2016	Cohort	Short-term	China	28.9 + 2.0; 29.0 + 1.6	1244 ± 321 g; 1208 ± 266 g	402 (162/240)	Respiratory failure	Blank	5	5
Hasan	2017	RCT	Short-term and long-term	US and Canada	25.6 ± 1.4; 25.6 ± 1.4	724 ± 160 g; 750 ± 164 g	451 (229/222)	Premature	Placebo (nitrogen)	20	20
Chandra-sekharan	2017	Cohort	Short-term and long-term	American	26 ± 4; 26 ± 2	912 ± 517 g; 892 ± 243 g	1023 (93/930)	Respiratory failure /PH	Blank	–	–
Carey	2018	Cohort	Short-term	American	25.7 ± 1.9; 25,6 ± 1.9	840 ± 280 g; 820 ± 280 g	1942 (971/971)	RDS	–	–	–
Collura	2018	Cohort	Short-term	American	–	–	1116 (558/558)	RDS and PPHN	–	–	–
Ellsworth	2018	Cohort	Short-term	American	26.7 ± 1.7; 26.7 ± 1.6	1020 ± 300 g; 1030 ± 270 g	302 (151/151)	Pulmonary hypoplasia	–	–	–
Udland	2019	Cohort	Short-term	American	25.9 ± 1.9; 26.1 ± 1.9	870 ± 290 g; 860 ± 280 g	1529 (660/869)	RDS and PH	–	–	–
Chandra-sekharan	2020	Cohort	Short-term	American	24.4 ± 1.2; 24.4 ± 1.1	652.4 ± 150.8 g; 667.5 ± 143.3 g	1732 (338/1394)	Pulmonary hypoplasia	–	–	–
Venkata	2024	Cohort	Short-term and long-term	Canada	25 (24,27); 26 (25,28)	793 ± 252 g; 921 ± 240 g	4477 (414/4063)	Respiratory failure	–	–	–
Siljehav	2024	Cohort	Short-term and long-term	European countries	27.1 (25.4,29.1); 27.0 (25.4,28.7)	987 ± 406 g; 950 ± 385 g	568 (284/284)	Premature	–	–	–
Mirza	2025	RCT	Short-term	American	26.25 ± 4.2; 26.2 ± 4.4	914 ± 481 g; 966 ± 504 g	32 (16/16)	PH	Placebo	20	–

GA: gestational age; BW: birth weight; T/C: Treatment Group/Control Group; RCT: Randomized controlled trial; Cohort: cohort study; RDS: Respiratory distress syndrome; PH: Pulmonary hypoplasia; PPHN: persistent pulmonary hypertension of the newborn; -:Data not reported in the primary studies

### Primary outcome

#### Death before discharge

**Fig. 2:**
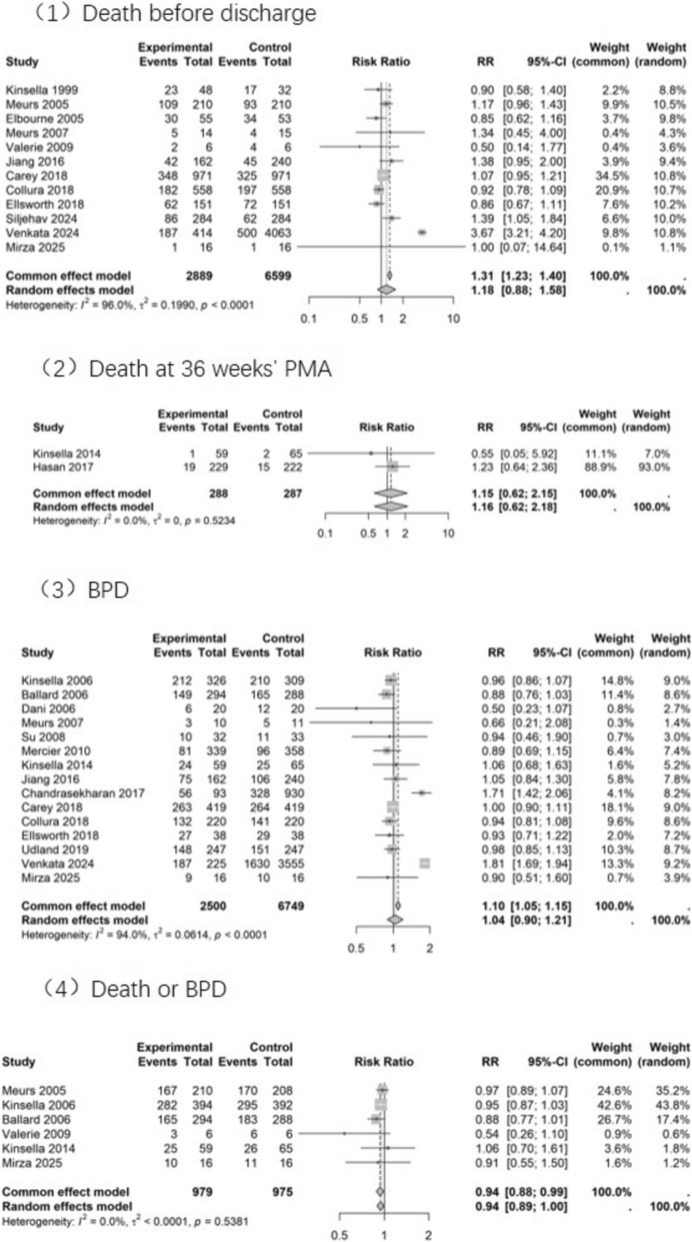
Forest plots of primary outcomes

A total of twelve studies reported this outcome, revealing no statistically significant difference between the iNO and control groups (Fig. 2 and Online Resource 3). Subgroup analyses based on study design and specific diseases of preterm infants were conducted but showed no statistical significance (Online Resource 4). In the RCT subgroup (4 studies), the outcome was assessed as having a serious risk of bias and very serious imprecision, resulting in a very low quality of evidence. Similarly, in the non-RCT subgroup (5 studies), the evidence was rated as very low quality due to a very serious risk of bias and serious imprecision (Table 2).

**Table 2:** GRADE Working Group grades of evidence of subgroup of primary outcomes

Outcomes	Anticipated absolute effects^*^ (95% CI)		Relative effect (95% CI)	№ of participants (studies)	Certainty of the evidence (GRADE)	Comments
	Risk with control	Risk with iNO				
Death before discharge RCT	457 per 1000	**489per 1000** (416 to 571)	**RR 1.07** (0.91 to 1.25)	669 (5 RCTs)	⨁◯◯◯ Very low^a,b,c^	The evidence is very uncertain about the effect of iNO on death before discharge
Death before discharge Non-RCT	192 per 1000	**196 per 1000** (179 to 213)	**RR 1.02** (0.93 to 1.11)	8819 (7 non-randomised studies)	⨁◯◯◯ Very low^c,d,f^	iNO may have no effect on death before discharge
Death at 36 week’s PMA RCT	59 per 1000	**68 per 1000** (37 to 127)	**RR 1.15** (0.62 to 2.15)	575 (2 RCTs)	⨁⨁◯◯ Low^b,c^	INO may result in little to no difference in death at 36 week’s PMA RCT
BPD RCT	485 per 1000	**442 per 1000** (408to 481)	**RR 0.91** (0.84 to 0.99)	2196 (8RCTs)	⨁⨁⨁◯ Moderate^e^	iNO probably results in a large reduction in BPD
BPD Non-RCT	469 per 1000	**502 per 1000** (427 to 596)	**RR 1.07** (0.91 to 1.27)	7053 (7 non-randomised studies)	⨁◯◯◯ Very low^d,f^	The evidence is very uncertain about the effect of iNO on BPD
Death or BPD-RCT	707 per 1000	**664 per 1000** (629 to 707)	**RR 0.94** (0.89 to 1.00)	1942 (5RCTs)	⨁⨁⨁⨁ High	iNO results in large reduction in Death or BPD

^*^The risk in the intervention group (and its 95% confidence interval) is based on the assumed risk in the comparison group and the **relative effect** of the intervention (and its 95% CI). **The “Comments” column**: It is a conclusion drawn by integrating the results and the quality of the evidence. *GRADE Working Group grades of evidence* **High certainty:** we are very confident that the true effect lies close to that of the estimate of the effect. **Moderate certainty:** we are moderately confident in the effect estimate: the true effect is likely to be close to the estimate of the effect, but there is a possibility that it is substantially different. **Low certainty:** our confidence in the effect estimate is limited: the true effect may be substantially different from the estimate of the effect. **Very low certainty:** we have very little confidence in the effect estimate: the true effect is likely to be substantially different from the estimate of effect

CI: confidence interval; RR: risk ratio

^a^One study lacks of allocation concealment and blinding

^b^Total number of occurrences of the outcome was small

^c^95%CI was large

^d^All studies lack of allocation concealment and blinding

^e^One study lacks of blinding (Su 2008)

^f^P value of the test of heterogeneity less than 0.01

#### Death at 36 weeks’ PMA

Two studies reported this outcome, and no statistically significant difference was observed (Fig. 2 and Online Resource 3). Both studies were RCTs, and the quality of evidence was rated as low due to very serious imprecision (Table 2).

#### BPD

Fifteen studies explored this outcome, and no statistically significant difference was observed (Fig. 2 and Online Resource 3). However, subgroup analysis of eight RCTs revealed a reduced incidence of BPD in preterm infants with respiratory disease following iNO treatment compared to the control group (RR 0.91, 95% CI 0.84 to 0.99; 8 studies, 2196 infants; *I*^*2*^ = 0%), as detailed in Online Resource 4. The evidence was rated as moderate quality due to a serious risk of bias in the RCT subgroup. In the non-RCT subgroup (7 studies), the evidence was rated as very low quality because of a very serious risk of bias and serious inconsistency (Table 2).

#### Death or BPD

Six studies reported this outcome (Fig. 2 and Table 1), demonstrating that iNO reduced the incidence of death or BPD compared to controls (RR 0.94, 95% CI 0.88 to 0.99, 6 studies, 1954 infants, *I*^2^ = 0%). Subgroup analysis of 4 RCTs similarly showed a reduced incidence of death or BPD, though this did not reach statistical significance (RR 0.94, 95% CI 0.89 to 1.00, 5 studies, 1942 infants, *I*^2^ = 0%), as shown in Online Resource 4. The quality of evidence was rated as high (Table 2).

### Secondary outcomes

All secondary outcomes are shown in Online Resource 5.

#### Pulmonary parameters

Two RCTs reported OI and PaO_2_ outcomes. At baseline, following the first 30-min treatment with 5 ppm iNO, significant improvements were observed compared to controls: OI decreased (SMD -0.62, 95% CI -0.81 to -0.43, 2 studies, 441 infants, *I*^*2*^ = 5.1%) and PaO₂ levels increased(SMD 0.68, 95% CI 0.49 to 0.87, 2 studies, 441 infants, *I*^*2*^ = 0%).Furthermore, more patients in the iNO group achieved a PaO₂ increase > 20 mmHg (RR 3.17, 95% CI 2.32 to 4.33, 2 studies, 441 infants, *I*^*2*^ = 0%),while fewer showed minimal improvement (< 10 mmHg increase; RR 0.39, 95% CI 0.31 to 0.48, 2 studies, 441 infants, *I*^*2*^ = 0%). These results are presented in Table 3.

**Table 3:** Summary of significant secondary outcomes

Outcomes	No. of studies	*I*^2^ (%)	Model	SMD	RR	95% CI lower	95% CI upper
Change in OI after 30 min (5 ppm)	2	5	Fixed	– 0.62	/	– 0.81	– 0.43
Change in PaO2 after 30 min (5 ppm)	2	0	Fixed	0.68	/	0.49	0.87
Increase larger than 20 mmHg in PaO2 after 30 min (5 ppm)	2	0	Fixed	3.17	/	2.32	4.32
Increase lower than 10 mmHg in PaO2 after 30 min (5 ppm)	2	0	Fixed	0.39	/	0.31	0.48
Supplemental oxygen at 1 ear corrected age	3	0	Fixed	/	0.79	0.65	0.96
ROP	17	20	Fixed	/	1.08	1.01	1.16
Head circumference at 1 ear corrected age	2	11	Fixed	/	– 0.17	– 0.32	– 0.02

RR: risk ratio; SMD: standardized mean difference; 95% CI: 95% confidence interval; Model: Statistical models for effect size combination; Random: random-effects model; Fixed: fixed-effects model

#### Respiratory outcomes

No significant differences were observed between the iNO and control groups for pulmonary hemorrhage (RR 1.06, 95% CI 0.79 to 1.43, 7 studies, 2447 infants, *I*^*2*^ = 0.0%) or air leakage (RR 0.88, 95% CI 0.71 to 1.08, 8 studies, 2836 infants, *I*^*2*^ = 0.0%). However, iNO administration significantly reduced the need for supplemental oxygen at 1 year corrected age compared to controls (RR 0.79, 95% CI 0.65 to 0.96, 3 studies, 1259 infants, *I*^*2*^ = 0.0%), as detailed in Table 3.

#### Neurodevelopmental outcomes

Regarding short-term neurodevelopmental outcomes, a significant difference was observed only in the meta-analysis of ROP incidence (RR 1.08, 95% CI 1.01 to 1.16, 17 studies, 7220 infants, *I*^2^ = 20%). However, no statistically significant differences were found for either ROP requiring treatment or stage III and above ROP, indicating that iNO administration did not affect severe ROP. Other short-term neurodevelopmental outcomes—including IVH, IVH (Grade 3–4), PVL, and Grade 3 or 4 IVH or PVL—similarly showed no significant differences between groups (Online Resource 5).

For long-term neurodevelopmental outcomes, a slight reduction in head circumference was observed in infants treated with iNO (SMD − 0.17, 95% CI − 0.32 to − 0.02, 2 studies, 699 infants, *I*^2^ = 11%; Table 3). However, no statistically significant differences were found in other outcomes, including: CP at 18–26 months corrected age, NDI at 18–24 months corrected age, loss of vision at 18–26 months corrected age, and visual impairment at 18–26 months corrected age (Online Resource 5).

#### Other outcomes

There were no statistically significant differences in the outcomes including NEC, symptomatic PDA, vasopressor use, postnatal steroids and duration of hospitalization in survivors (Online Resource 5).

### Bias

We assessed the risk of bias for both RCTs and non-randomized observational studies. Overall, all included studies demonstrated relatively low risk of bias (Online Resource 6). Publication bias was evaluated using funnel plots (Online Resource 7), Begg's test and Egger's test (Online Resource 3), of primary outcomes, with no significant biases detected.

### Heterogeneity and sensitivity analysis

Substantial heterogeneity was observed for the outcomes of death before discharge (*I*^*2*^ = 96%) and BPD (*I*^*2*^ = 70.0%). Through sensitivity analysis involving sequential exclusion of individual studies, we identified the studies by Chandrasekharan et al. and Venkata et al. as the primary sources of heterogeneity (Online Resource 8).

## Discussion

This meta-analysis examined the evidence regarding iNO use in preterm infants with specific respiratory diseases. Overall, we observed a reduction in the incidence of death or BPD, as well as a decreased incidence of BPD in RCT subgroup. Additionally, iNO was associated with improvements in oxygenation parameters (OI and PaO₂) and a reduced need for supplemental oxygen at 1 year of corrected age. However, the iNO group showed an increased occurrence of ROP and a smaller head circumference compared to the control group, though no significant differences were observed in severe ROP or other short- and long-term neurodevelopmental outcomes. Furthermore, there were no significant differences in other neonatal morbidities or adverse events.

Our study found no significantly statistical difference in death before discharge or death at 36 weeks’ PMA. According to the GRADE assessment, the certainty of evidence for death before discharge in the RCT subgroup was rated as very low due to: lack of allocation concealment and blinding in one trail, few outcome events, and wide 95%CI in RCT. In the non-RCT subgroup of death before discharge, the certainty of evidence was also rated as very low due to wide 95%CI, lack of allocation concealment and blinding and high heterogeneity. The certainty of evidence for death at 36 weeks’ PMA was rated as low due to the small number of outcome events and wide 95% CI in the RCT subgroup. These findings suggest that the evidence remains inconclusive regarding the effect of iNO on mortality outcomes in preterm infants. Numerous studies have demonstrated that iNO therapy does not reduce mortality in preterm infants. A Cochrane meta-analysis examining iNO use in preterm infants (< 35 weeks gestation) with respiratory failure reached the same conclusion [17]. Similarly, a meta-analysis of 11 RCTs found no significant effect of iNO treatment on preterm infant mortality [18]. Current evidences show no increased risk of preterm mortality associated with iNO therapy. Future large-scale, high-quality RCTs are warranted to address this knowledge gap, particularly given the low certainty of existing evidence (GRADE: low to very low).

Our meta-analysis included 15 studies reporting BPD outcomes. While the overall analysis showed no significant difference between iNO and control groups, subgroup analysis of RCTs demonstrated that iNO significantly reduced BPD incidence. According to the GRADE assessment, the certainty of evidence for BPD was rated as moderate in the RCT subgroup. This rating reflects that while one trial lacked allocation blinding, the true effect likely approximates the estimated effect, though additional research might modify this conclusion. In contrast, the non-RCT group evidence was rated as very low certainty due to absence of allocation concealment, lack of blinding, and significant heterogeneity. These limitations indicate substantial uncertainty about iNO's effect on BPD in non-RCT studies. The observed discrepancy between two subgroups likely arises from differential effects between RCTs and non-RCT study designs. A Cochrane meta-analysis reported findings partially inconsistent with our results [17]. That study stratified infants into three subgroups: those < 3 days old based on oxygenation criteria, those ≥ 3 days old based on BPD risk, and intubated preterm infants. Only the second subgroup demonstrated a marginal BPD reduction (RR 0.91, 95% CI 0.83–1.01). These discrepancies may arise from: (1) population differences: Our study included all preterm infants (though mostly were < 34 weeks), versus their strict < 35 weeks. (2) Disease spectrum: We incorporated various respiratory disorders (including respiratory failure), while they focused solely on surfactant-treated respiratory failure cases. These findings suggest that when considering iNO therapy for preterm infants, clinicians should evaluate not only gestational age/weight but also specific respiratory pathology, and optimal treatment timing. Notably, a recent meta-analysis in preterm infants ≤ 34 weeks with HRF found iNO reduced BPD incidence [19] and an umbrella review similarly reported iNO's association with decreased BPD risk [20]. The observed BPD reduction in our RCT subgroup appears robust (narrow 95% CI not crossing unity), suggesting iNO may substantially decrease BPD risk in preterm infants. However, this interpretation requires caution given non-replication in non-RCT studies, and inconsistent findings across previous research.

Our analysis demonstrated that iNO could reduce the incidence of the composite outcome of death or BPD. Existing literature on this outcome in preterm infants with respiratory disease has shown inconsistent results. While the Cochrane meta-analysis reported divergent findings [17]- likely due to the methodological and population differences noted previously—an umbrella review similarly found no significant reduction in death or BPD with iNO therapy [20]. However, other meta-analyses have reported beneficial effects, showing iNO effectively decreased this composite outcome in preterm infants ≤ 34 weeks [21] and those with HRF in preterm infants ≤ 34 weeks [19]. Notably, our RCT subgroup analysis revealed favorable outcomes compared to controls. The GRADE assessment indicated high-quality evidence for this subgroup, suggesting the estimated effect closely approximates the true effect. Although the reduction did not reach statistical significance (RR 0.94, 95% CI 0.89 to 1.00), this finding aligned with the overall analysis incorporating all study designs. These results support considering iNO therapy for preterm infants, with our data suggesting iNO may substantially reduce death or BPD incidence. Moreover, the observed benefit in the composite outcome “death or BPD” appears to be primarily driven by iNO's protective effect against BPD, given both the absence of significant mortality reduction and the significant decrease in BPD incidence observed in the RCT subgroup analysis.

Our meta-analysis demonstrated that iNO therapy is associated with distinct improvements: improved OI, increased PaO₂, and reduced need for supplemental oxygen at 1 year of corrected age. However, the clinical significance of these independent improvements in predicting these prognosis remains uncertain due to the limited sample size.

The potential impact of iNO on IVH incidence warrants particular attention, given early evidence that NO may interfere with platelet adhesion to vascular endothelium and platelet aggregation in acute respiratory distress syndrome (ARDS) patients [22, 23]. Existing studies present conflicting findings: Marks et al. reported that iNO reduces severe acute brain injury incidence and improves neurodevelopmental outcomes [24], while Keith et al. observed a non-significant 20% increase in severe IVH associated with early iNO rescue therapy for impaired oxygenation [17]. Conversely, Lisa et al. found no statistically significant effect of iNO treatment on severe neurological events detected through imaging [25]. In our meta-analysis, no significant difference was found in the incidence of IVH or grade 3 or 4 IVH. These conflicting results highlight the need for further large-scale, well-designed studies to clarify the neurological safety profile of iNO in preterm infants.

Our results indicated no increase in other short- or long-term adverse neurological outcomes except for an increased incidence of ROP and smaller head circumference. The iNO group showed a slightly higher incidence of ROP compared to controls. ROP development is associated with hyperoxygenation effects on the developing retinal vasculature in preterm infants [26, 27]. Furthermore, rapid fluctuations in oxygen levels may contribute to the development of retinopathy of ROP, with these fluctuations being more likely to induce ROP occurrence at higher inspired oxygen concentrations compared to lower ones. [28]. Most infants requiring iNO therapy have severe respiratory diseases, typically requiring high fractional inspired oxygen. As our study found, the PO_2_ increases rapidly after NO inhalation, which may explain the association with retinopathy of ROP [29]. However, the use of iNO was not associated with an increased risk of ROP requiring treatment, long-term visual impairment, or blindness, suggesting no adverse effects on long-term ocular development. We found a smaller head circumference at 1 year corrected age as well. The two original studies included in the meta-analysis, with follow-up assessments at 1 year and 2 years of corrected age respectively, found no differences in neurodevelopmental abnormalities, including cognitive development, motor development, seizures, and visual and auditory development [30, 31]. Additionally, another study with follow-up until 5 years of corrected age reported comparable findings [32]. Due to the small sample size of head circumference reduction, as well as no difference in final neurological follow-up outcomes, we considered it an unimportant aspect.

Significant heterogeneity was observed in the outcomes of death before discharge and BPD. Our analysis identified the studies by Chandrasekharan et al. [33] and Venkata et al. [34] as the primary sources of this heterogeneity. Regarding the Chandrasekharan et al. study, we identified several potential contributors to heterogeneity: Firstly, the study design was retrospective rather than prospective. Secondly, the enrolled population consisted exclusively of extremely preterm infants (birth weight < 1000 *g* and gestational age < 28 weeks), a subgroup for which current evidence regarding iNO's potential benefits remains inconclusive. Thirdly, the study groups exhibited significant baseline imbalances, with the iNO group showing substantially higher rates of oligohydramnios and prolonged rupture of membranes (both ≥ 18 h and ≥ 120 h). These clinical factors are risk markers for both pulmonary hypoplasia and adverse neonatal outcomes, including BPD, in preterm populations. Regarding the Venkata et al. study, the primary source of heterogeneity stemmed from significant baseline imbalances between the iNO and control groups. Specifically, the iNO group demonstrated lower rates of antenatal steroid administration, reduced gestational age and birth weight, more small-for-gestational-age infants, higher incidence of prolonged premature rupture of membranes (> 24 h).These demographic and clinical characteristics represent potential risk factors for adverse neonatal outcomes in preterm populations, possibly contributing to worse prognostic trends in the iNO treatment group. More importantly, subgroup analyses restricted to RCTs demonstrated no significant heterogeneity for either death before discharge or BPD outcomes. This indicates that the observed heterogeneity in the overall analysis did not materially affect these primary outcomes when considering only high-quality randomized evidence.

Compared with previous meta-analyses and systematic reviews, this study incorporates the most recent evidence, extends subgroup analyses of iNO efficacy in preterm populations, and importantly, provides novel data on iNO's long-term neurodevelopmental outcomes (e.g. CP, neurodevelopmental problems, visual impairment, hearing impairment). Furthermore, our inclusion of both randomized controlled trials and cohort studies, along with comprehensive data collection, strengthens the robustness of our meta-analytic findings. However, several limitations should be acknowledged. Firstly, the iNO dosage and timing subgroups are not processed properly because of the diverse designs of the dosage range and timing of therapy in the trials. Secondly, inherent methodological diversity—including variations in study designs (e.g., differing randomization methods in trials and control selection approaches in observational studies), intervention protocols (divergent iNO initiation criteria and dose escalation schemes), and outcome measurements (inconsistent BPD diagnostic criteria)—limited the analyzable data despite comprehensive collection efforts. Thirdly, the interpretation of observational study findings warrants caution due to susceptibility to residual confounding (e.g., baseline disparities in gestational age, birth weight, or disease severity), as unmeasured variables (e.g., center-specific treatment protocols) may still confound the results despite some studies implementing statistical adjustments. Fourth, the interpretation of death at 36 weeks’ PMA, pulmonary parameters, supplemental oxygen at 1 year corrected age, IVH (Grade 3–4), Grade 3 or 4 IVH or PVL, stage III and above ROP, head circumference at 1 year corrected age, NDI at 18 to 24 months corrected age, Vasopressor use, Duration of hospitalization in survivors should be cautious due to limited data(< 5 studies). Finally, only English articles were included in this article, thus relevant studies published in other languages may have been missed.

Our findings demonstrate that iNO may reduce the incidence of death or BPD and BPD alone in preterm infants, suggesting its potential value in preventing this chronic lung disease. However, clinicians should carefully weigh these potential benefits against the existing limitations and the modestly increased risk of ROP, as previously discussed. Future research should prioritize large, well-designed RCTs to determine optimal iNO administration protocols (including timing and dosage) for specific preterm infant subgroups, particularly extremely low birth weight (ELBW) infants. These studies should incorporate long-term follow-up to comprehensively assess neurodevelopmental and respiratory outcomes.

## Conclusion

This analysis showed beneficial effects of iNO in reducing the risk of death or BPD and a probable reduction in BPD incidence in preterm infants with respiratory diseases. The treatment exhibited a favorable safety profile across evaluated outcomes. However, given current evidence limitations, future high-quality studies are needed to further investigate iNO's effects, particularly its long-term neurodevelopmental impacts, particularly ELBW infants.

## What is known?

In recent years, inhaled nitric oxide (iNO) has been used in term or near-term infants with respiratory distress. It has reduced mortality or the need for extracorporeal membrane oxygenation (ECMO) in neonates with persistent pulmonary hypertension of the newborn (PPHN) and hypoxic respiratory failure (HRF).

The United States Food and Drug Administration approved iNO for PPHN treatment in infants > 34 weeks' gestational age at birth.

## What is new?

Currently, the use of iNO in preterm infants ≤ 34 weeks gestation is off-label and mostly limited to emergency rescue mostly, with unclear efficacy.

Published data indicate that the effectiveness and safety of iNO for treating respiratory disease in preterm infants remain controversial.

While iNO may reduce the incidence of the composite outcome (death or bronchopulmonary dysplasia, BPD) as well as BPD alone, it shows no significant effect on other morbidities or adverse events.

## Supplementary Information

Supplementary Material 1.

Supplementary Material 2.

Supplementary Material 3.

Supplementary Material 4.

Supplementary Material 5.

Supplementary Material 6.

Supplementary Material 7.

Supplementary Material 8.

## Data Availability

The basic data for this article can be found in the article and the online supplementary material. The raw data will be shared with you when necessary.

## Abbreviations

RR: Risk ratio
95%CI: 95% Confidence interval
BW: Birth weight
BPD: Bronchopulmonary dysplasia
CP: Cerebral palsy
ECMO: Extracorporeal membrane oxygenation
ELBW: Extremely low birth weight
HRF: Hypoxic respiratory failure
iNO: Inhaled nitric oxide
IVH: Intraventricular hemorrhage
MAS: Meconium aspiration syndrome
NEC: Necrotizing enterocolitis
NDI: Neurodevelopmental impairment
OI: Oxygenation index
PaO_2_: Partial pressure of oxygen
PDA: Patent ductus arteriosus
PVL: Periventricular leukomalacia
PPHN: Persistent pulmonary hypertension of the newborn
PH: Pulmonary hypoplasia
RCT: Randomized controlled trial
RDS: Respiratory distress syndrome
ROP: Retinopathy of prematurity
RR: Risk ratio
SMD: Standardized mean difference
