# Supplementary material for: Inhaled nitric oxide in preterm infants with respiratory disease: a systematic review and meta-analysis
Source: Eur J Med Res. 2025 Aug 29;30:821. doi: 10.1186/s40001-025-03008-1 (PMC12395824; doi:10.1186/s40001-025-03008-1)
Supplement: Supplementary file 1 — Supplementary Material 1. [file 40001_2025_3008_MOESM1_ESM.pdf]

Appendix. File 6. Sensitivity analysis

Article title: Inhaled nitric oxide in preterm infants with respiratory disease: a systematic review and meta-analysis

Journal name: European Journal of Medical Research.

Author names: Kai Zhouc, Weipeng Xua,Danrui Li, CheokUn Laoa, Shiqian Zouc, Shixian Liuc, Bingxiao Lia,Fangfang Zengb, Sui Zhub, Shasha Hana.

Affiliation and e-mail address of the corresponding author: Department of Neonatology and Pediatrics, The First Affiliated Hospital of Jinan University, Guangzhou, Guangdong, China;hanssha888@163.com.

(A) ***Death before discharge***

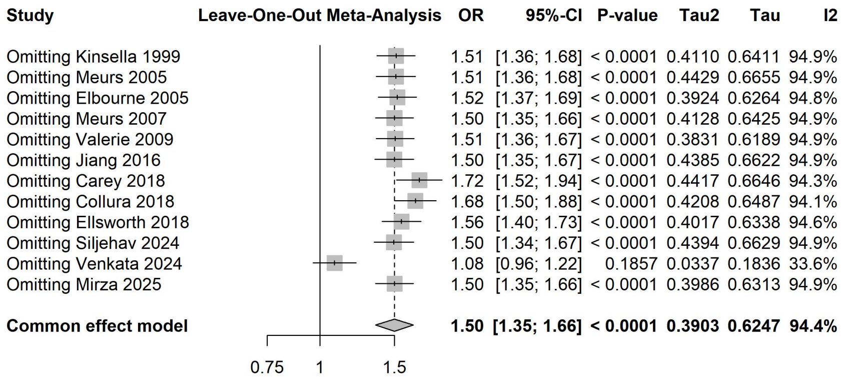

(B) ***Death at 36 weeks' PMA***

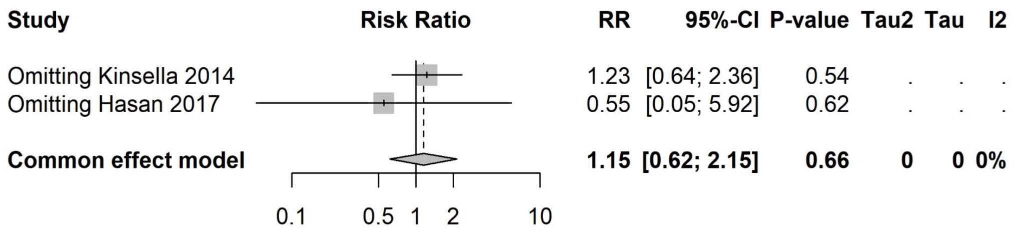

(C) ***BPD***

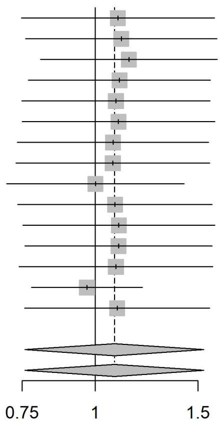

Study	OR	95%-CI	P-value	Tau2	Tau	I2
Omitting Kinsella 2006	1.09	[0.75; 1.60]	0.6449	0.3970	0.6301	88.7%
Omitting Ballard 2006	1.11	[0.76; 1.62]	0.5924	0.3873	0.6223	88.3%
Omitting Dani 2006	1.14	[0.81; 1.62]	0.4543	0.3380	0.5814	88.6%
Omitting Meurs 2007	1.10	[0.77; 1.58]	0.6021	0.3693	0.6077	88.9%
Omitting Su 2008	1.09	[0.75; 1.57]	0.6654	0.3868	0.6219	89.0%
Omitting Mercier 2010	1.10	[0.75; 1.60]	0.6365	0.3956	0.6289	88.7%
Omitting Kinsella 2014	1.07	[0.74; 1.56]	0.7155	0.3962	0.6294	89.0%
Omitting Jiang 2016	1.07	[0.73; 1.57]	0.7218	0.4029	0.6347	89.0%
Omitting Chandrasekharan 2017	1.00	[0.71; 1.42]	0.9950	0.3240	0.5692	87.2%
Omitting Carey 2018	1.08	[0.74; 1.59]	0.6897	0.4029	0.6348	88.8%
Omitting Collura 2018	1.10	[0.75; 1.60]	0.6301	0.3939	0.6276	88.7%
Omitting Ellsworth 2018	1.10	[0.76; 1.59]	0.6248	0.3821	0.6182	88.9%
Omitting Udland 2019	1.09	[0.74; 1.59]	0.6748	0.4005	0.6328	88.9%
Omitting Venkata 2024	0.97	[0.78; 1.20]	0.7708	0.0883	0.2971	57.2%
Omitting Mirza 2025	1.09	[0.76; 1.57]	0.6414	0.3785	0.6153	89.0%
**Common effect model**	**1.08**	**[0.76; 1.53]**	**0.6738**	**0.3644**	**0.6037**	**88.1%**
**Random effects model**	**1.08**	**[0.76; 1.53]**	**0.6738**	**0.3644**	**0.6037**	**88.1%**

(D) *Death or BPD*

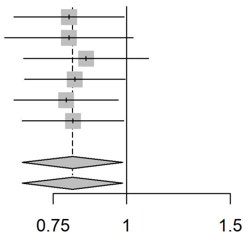

Study	OR	95%-CI	P-value	Tau2	Tau	I2
Omitting Meurs 2005	0.80	[0.64; 0.99]	0.0400	< 0.0001	0.0015	0%
Omitting Kinsella 2006	0.80	[0.62; 1.03]	0.0775	< 0.0001	0.0019	0%
Omitting Ballard 2006	0.85	[0.67; 1.09]	0.2018	< 0.0001	0.0013	0%
Omitting Valerie 2009	0.82	[0.67; 0.99]	0.0436	0	0	0%
Omitting Kinsella 2014	0.79	[0.64; 0.97]	0.0232	< 0.0001	0.0023	0%
Omitting Mirza 2025	0.81	[0.66; 0.99]	0.0378	< 0.0001	0.0006	0%
**Common effect model**	**0.81**	**[0.66; 0.99]**	**0.0350**	**< 0.0001**	**0.0003**	**0%**
**Random effects model**	**0.81**	**[0.66; 0.99]**	**0.0350**	**< 0.0001**	**0.0003**	**0%**
