# Supplementary material for: Inhaled nitric oxide in preterm infants with respiratory disease: a systematic review and meta-analysis
Source: Eur J Med Res. 2025 Aug 29;30:821. doi: 10.1186/s40001-025-03008-1 (PMC12395824; doi:10.1186/s40001-025-03008-1)
Supplement: Supplementary file 2 — Supplementary Material 2. [file 40001_2025_3008_MOESM2_ESM.pdf]

Appendix. File 8. Databases search queries

Article title: Inhaled nitric oxide in preterm infants with respiratory disease: a systematic review and meta-analysis

Journal name: European Journal of Medical Research.

Author names: Kai Zhouc, Weipeng Xua,Danrui Li, CheokUn Laoa, Shiqian Zouc, Shixian Liuc, Bingxiao Lia,Fangfang Zengb, Sui Zhub, Shasha Hana.

Affiliation and e-mail address of the corresponding author: Department of Neonatology and Pediatrics, The First Affiliated Hospital of Jinan University, Guangzhou, Guangdong, China;hanssha888@163.com.

Pubmed search query

((((("Nitric Oxide"[Mesh] AND Humans[Mesh])) OR ((((((((((((Oxide[Title/Abstract]) AND Nitric[Title/Abstract] AND Humans[Mesh])) OR ((Monoxide[Title/Abstract]) AND Nitrogen[Title/Abstract] AND Humans[Mesh])) OR ((Nitric Oxide[Title/Abstract]) AND Endothelium-Derived[Title/Abstract] AND Humans[Mesh])) OR ((Nitric Oxide[Title/Abstract]) AND Endothelium Derived[Title/Abstract] AND Humans[Mesh])) OR ((Nitrate Vasodilator[Title/Abstract]) AND Endogenous[Title/Abstract] AND Humans[Mesh])) OR ((Vasodilator[Title/Abstract]) AND Endogenous Nitrate[Title/Abstract] AND Humans[Mesh])) OR ((Monoxide[Title/Abstract]) AND Mononitrogen[Title/Abstract] AND Humans[Mesh])) OR Nitrogen Monoxide[Title/Abstract]) OR Endothelium-Derived Nitric Oxide[Title/Abstract]) OR Endogenous Nitrate Vasodilator[Title/Abstract]) OR Mononitrogen Monoxide[Title/Abstract]) AND Humans[Mesh])) AND Humans[Mesh])) AND (((((((("Premature Birth"[Mesh] AND Humans[Mesh])) OR ((((((Birt*[Title/Abstract]) AND Premature[Title/Abstract] AND Humans[Mesh])) AND ((Birt*[Title/Abstract]) AND Preterm[Title/Abstract] AND Humans[Mesh])) AND

Premature Birt*[Title/Abstract]) AND Preterm Birt*[Title/Abstract] AND Humans[Mesh])) AND Humans[Mesh])) OR (((("Infant, Premature"[Mesh] AND Humans[Mesh])) OR ((((((((Prematurity[Title/Abstract]) AND Neonatal[Title/Abstract] AND Humans[Mesh])) OR ((Infan*[Title/Abstract]) AND Preterm[Title/Abstract] AND Humans[Mesh])) OR ((Infan*[Title/Abstract]) AND Premature[Title/Abstract] AND Humans[Mesh])) OR Premature Infan*[Title/Abstract]) OR Preterm Infan*[Title/Abstract]) OR Neonatal Prematurity[Title/Abstract]) AND Humans[Mesh])) AND Humans[Mesh])) OR (((("Infant, Extremely Low Birth Weight"[Mesh] AND Humans[Mesh])) OR (((Extremely Low Birth Weight Infant[Title/Abstract]) OR ELBW[Title/Abstract]) AND Humans[Mesh])) AND Humans[Mesh])) OR (((("Infant, Very Low Birth Weight"[Mesh] AND Humans[Mesh])) OR (((((((Infan*[Title/Abstract]) AND Very-Low-Birth-Weight[Title/Abstract] AND Humans[Mesh])) OR Very-Low-Birth-Weight Infan*[Title/Abstract]) OR Very Low Birth Weight Infan*[Title/Abstract]) OR Very Low Birth Weight[Title/Abstract]) OR VLBW[Title/Abstract]) AND Humans[Mesh])) AND Humans[Mesh])) AND Humans[Mesh]) Filters: **Humans**

Scopus search query

((TITLE-ABS-KEY (birt* AND premature) OR TITLE-ABS-KEY (premature AND birt*) OR TITLE-ABS-KEY (preterm AND birt*) OR TITLE-ABS-KEY (birt* AND preterm)) AND (LIMIT-TO (SUBJAREA , "MEDI")) OR (TITLE-ABS-KEY (infan* AND premature) OR TITLE-ABS-KEY (premature AND infan*) OR TITLE-ABS-KEY (preterm AND infan*) OR TITLE-ABS-KEY (infan* AND preter

m) OR TITLE-ABS-KEY (neonatal AND prematurity) OR TITLE-ABS-KEY (prematurity AND neonatal)) AND (LIMIT-TO (SUBJAREA , "MEDI")) OR (TITLE-ABS-KEY (infant AND extremely AND low AND birth AND weight) OR TITLE-ABS-KEY (extremely AND low AND birth AND weight AND infant) OR TITLE-ABS-KEY (elbw)) AND (LIMIT-TO (SUBJAREA , "MEDI")) OR (TITLE-ABS-KEY (very-low-birth-weight AND infan*) OR TITLE-ABS-KEY (infan* AND very-low-birth-weight) OR TITLE-ABS-KEY (very AND low AND birth AND weight AND infan*) OR TITLE-ABS-KEY (very AND low AND birth AND weight) OR TITLE-ABS-KEY (vlbw)) AND (LIMIT-TO (SUBJAREA , "MEDI")))

AND

(TITLE-ABS-KEY ("Nitric Oxide") OR TITLE-ABS-KEY ("Nitrogen Monoxide") OR TITLE-ABS-KEY ("Nitric Oxide Endothelium-Derived") OR TITLE-ABS-KEY ("Endothelium-Derived Nitric Oxide") OR TITLE-ABS-KEY ("Nitric Oxide Endothelium Derived") OR TITLE-ABS-KEY ("Endogenous Nitrate Vasodilator") OR TITLE-ABS-KEY ("Mononitrogen Monoxide") OR TITLE-ABS-KEY ("Vasodilator Endogenous Nitrate")) AND (LIMIT-TO (SUBJAREA , "MEDI"))

Web of science search query

(TS=(Premature Birth OR Infant, Premature OR Infant, Extremely Low Birth Weight OR Infant, Very Low Birth Weight) OR TI=(Birt*, Premature OR Premature Birt* OR Preterm

Birt* OR Birt*, Preterm OR Infants, Premature OR Premature Infan* OR Preterm Infan* OR Infan*, Preterm OR Neonatal Prematurity OR Prematurity, Neonatal OR Extremely Low Birth Weight Infant OR ELBW OR Very-Low-Birth-Weight Infan* OR Infan*, Very-Low-Birth-Weight OR Very Low Birth Weight Infan* OR Very Low Birth Weight OR VLBW)) AND (TS=Nitric Oxide OR TI=(Oxide, Nitric OR Nitrogen Monoxide OR Monoxide, Nitrogen OR Nitric Oxide, Endothelium-Derived OR Endothelium-Derived Nitric Oxide OR Nitric Oxide, Endothelium Derived OR Endogenous Nitrate Vasodilator OR Nitrate Vasodilator, Endogenous OR Vasodilator, Endogenous Nitrate OR Mononitrogen Monoxide OR Monoxide, Mononitrogen))

Cochrane search query

(((Premature Birt* OR Preterm Birt* OR Premature Infan* OR Preterm Infan* OR Neonatal Prematurity OR Very-Low-Birth-Weight Infan* OR Very Low Birth Weight Infan* OR Very Low Birth Weight OR Extremely Low Birth Weight Infan* OR ELBW OR VLBW):ti,ab,kw) AND ((Nitric Oxide OR Nitrogen Monoxide OR Nitrogen Monoxide OR Endothelium-Derived Nitric Oxide OR Endothelium Derived Nitric Oxide OR Endogenous Nitrate Vasodilator OR Mononitrogen Monoxide OR Nitric Oxide):ti,ab,kw) NOT (animals))
