# Supplementary material for: Inhaled nitric oxide in preterm infants with respiratory disease: a systematic review and meta-analysis
Source: Eur J Med Res. 2025 Aug 29;30:821. doi: 10.1186/s40001-025-03008-1 (PMC12395824; doi:10.1186/s40001-025-03008-1)

**Appendix. File 5. Funnel plots of primary outcomes**

**Article title:** Inhaled nitric oxide in preterm infants with respiratory disease: a systematic review and meta-analysis

**Journal name:** European Journal of Medical Research.

**Author names:** Kai Zhou, Weipeng Xu,Danrui Li, CheokUn Lao, Shiqian Zou, Shixian Liu, Bingxiao Li, Fangfang Zeng, Sui Zhu, Shasha Han.

**Affiliation and e-mail address of the corresponding author:**Department of Neonatology and Pediatrics, The First Affiliated Hospital of Jinan University, Guangzhou, Guangdong, China;hanssha888@163.com.

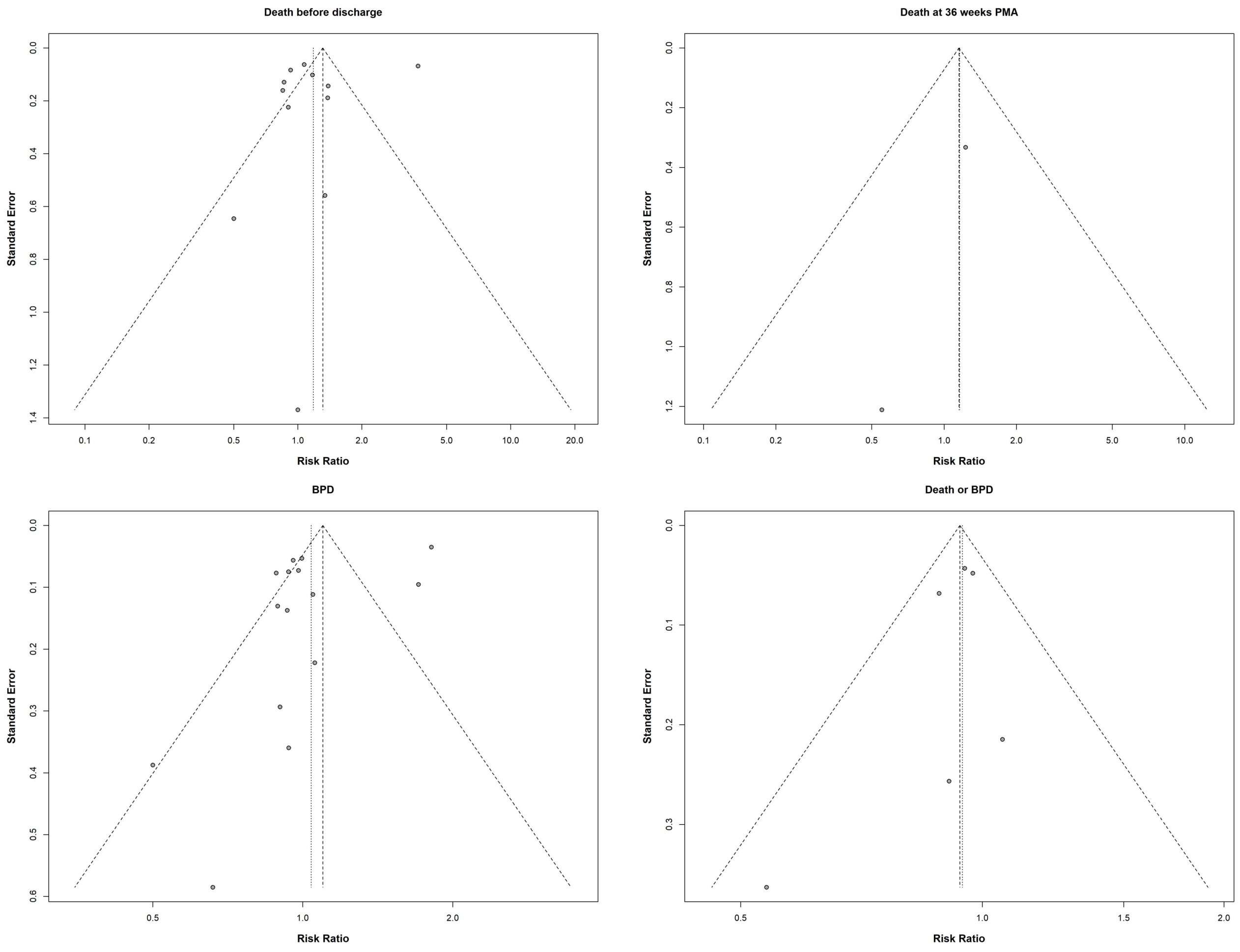

Supplement: Supplementary file 3 — Supplementary Material 3. [file 40001_2025_3008_MOESM3_ESM.pdf]
