# Supplementary material for: Inhaled nitric oxide in preterm infants with respiratory disease: a systematic review and meta-analysis
Source: Eur J Med Res. 2025 Aug 29;30:821. doi: 10.1186/s40001-025-03008-1 (PMC12395824; doi:10.1186/s40001-025-03008-1)
Supplement: Supplementary file 5 — Supplementary Material 5. [file 40001_2025_3008_MOESM5_ESM.pdf]

Appendix. File 3. Summary of meta-analysis and Publication bias of primary outcomes

Article title: Inhaled nitric oxide in preterm infants with respiratory disease: a systematic review and meta-analysis

Journal name: European Journal of Medical Research.

Author names: Kai Zhou, Weipeng Xu,Danrui Li, CheokUn Lao, Shiqian Zou, Shixian Liu, Bingxiao Li, Fangfang Zeng, Sui Zhu, Shasha Han.

Affiliation and e-mail address of the corresponding author: Department of Neonatology and Pediatrics, The First Affiliated Hospital of Jinan University, Guangzhou, Guangdong, China; hanssha888@163.com.

Outcomes	No. of studies	I^2	Model	RR	95% CI lower	95% CI upper	Publication bias
Death before discharge	12	96.0%	Random	1.18	0.88	1.58	Begg: 0.337<br>Egger: 0.443
Death at 36 weeks' PMA	2	0%	Fixed	1.15	0.62	2.15	/
BPD	15	94.0%	Random	1.04	0.90	1.21	Begg: 0.182<br>Egger: 0.077
Death or BPD	6	0.0%	Fixed	0.94	0.88	0.99	Begg:0.327<br>Egger: 0.370

RR: risk ratio,**95% CI**： 95% confidence interval, **Model:** Statistical models for effect size combination**: Random**: random-effects model, **Fixed**: fixed-effects model.

"Begg" represents the p-value from Begg's rank correlation test, **"Egger"** denote the p-values from Egger's regression tests. A non-significant result (P > 0.05) indicates a low probability of publication bias.
