# Supplementary material for: Inhaled nitric oxide in preterm infants with respiratory disease: a systematic review and meta-analysis
Source: Eur J Med Res. 2025 Aug 29;30:821. doi: 10.1186/s40001-025-03008-1 (PMC12395824; doi:10.1186/s40001-025-03008-1)

Appendix. File 2. Subgroups analysis

Article title: Inhaled nitric oxide in preterm infants with respiratory disease: a systematic review and meta-analysis

Journal name: European Journal of Medical Research.

Author names: Kai Zhou, Weipeng Xu,Danrui Li, CheokUn Lao, Shiqian Zou, Shixian Liu, Bingxiao Li, Fangfang Zeng, Sui Zhu, Shasha Han.

Affiliation and e-mail address of the corresponding author: Department of Neonatology and Pediatrics, The First Affiliated Hospital of Jinan University, Guangzhou, Guangdong, China;hanssha888@163.com.

(A) Subgroup analysis by study design

(1) Death before discharge

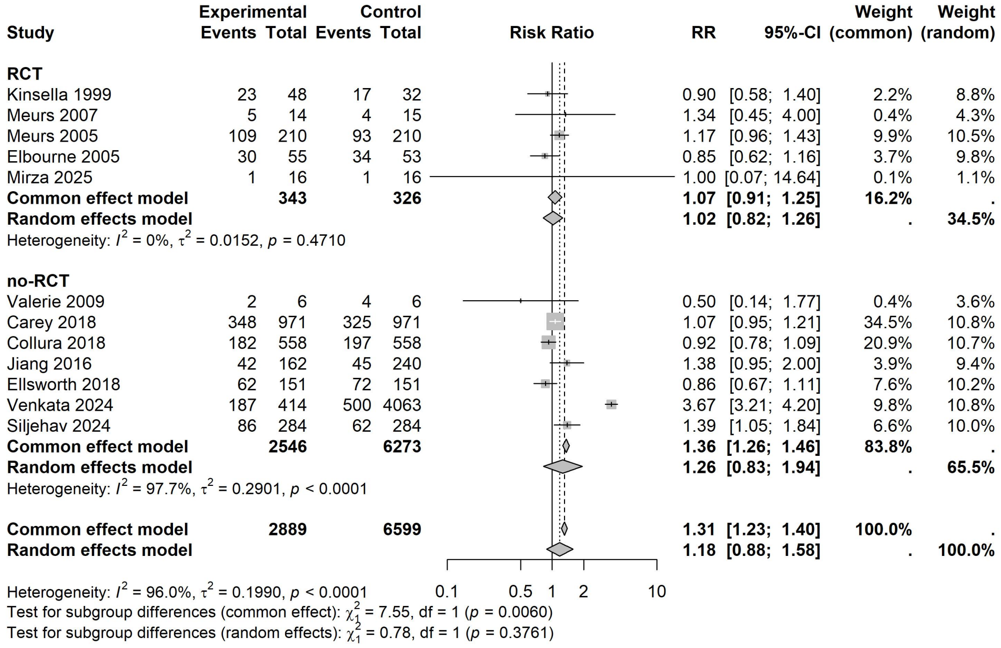

(2) Death at 36 weeks' PMA

Study	Experimental Events	Experimental Total	Control Events	Control Total	RR	95%-CI	Weight (common)	Weight (random)
**RCT**								
Kinsella 2014	1	59	2	65	0.55	[0.05; 5.92]	11.1%	7.0%
Hasan 2017	19	229	15	222	1.23	[0.64; 2.36]	88.9%	93.0%
**Common effect model**		**288**		**287**	**1.15**	**[0.62; 2.15]**	**100.0%**	**.**
**Random effects model**					**1.16**	**[0.62; 2.18]**	**.**	**100.0%**
Heterogeneity: I^2 = 0%, τ^2 = 0, p = 0.5234								
**Common effect model**		**288**		**287**	**1.15**	**[0.62; 2.15]**	**100.0%**	**.**
**Random effects model**					**1.16**	**[0.62; 2.18]**	**.**	**100.0%**

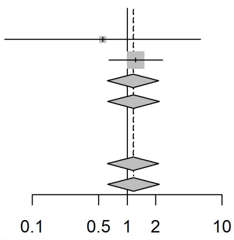

Heterogeneity: I^2 = 0.0%, τ^2 = 0, p = 0.5234
Test for subgroup differences (common effect): χ^2_0 = 0.00, df = 0 (p = NA)
Test for subgroup differences (random effects): χ^2_0 = 0.00, df = 0 (p = NA)

(3) BPD

Study	Experimental Events	Experimental Total	Control Events	Control Total	RR	95%-CI	Weight (common)	Weight (random)
**RCT**								
Meurs 2007	3	10	5	11	0.66	[0.21; 2.08]	0.3%	1.4%
Kinsella 2014	24	59	25	65	1.06	[0.68; 1.63]	1.6%	5.2%
Mercier 2010	81	339	96	358	0.89	[0.69; 1.15]	6.4%	7.4%
Su 2008	10	32	11	33	0.94	[0.46; 1.90]	0.7%	3.0%
Kinsella 2006	212	326	210	309	0.96	[0.86; 1.07]	14.8%	9.0%
Ballard 2006	149	294	165	288	0.88	[0.76; 1.03]	11.4%	8.6%
Dani 2006	6	20	12	20	0.50	[0.23; 1.07]	0.8%	2.7%
Mirza 2025	9	16	10	16	0.90	[0.51; 1.60]	0.7%	3.9%
**Common effect model**		**1096**		**1100**	**0.91**	**[0.84; 0.99]**	**36.8%**	**.**
**Random effects model**					**0.92**	**[0.85; 1.00]**	**.**	**41.2%**
Heterogeneity: I^2 = 0%, τ^2 = 0, p = 0.7790								
**no-RCT**								
Carey 2018	263	419	264	419	1.00	[0.90; 1.11]	18.1%	9.0%
Collura 2018	132	220	141	220	0.94	[0.81; 1.08]	9.6%	8.6%
Jiang 2016	75	162	106	240	1.05	[0.84; 1.30]	5.8%	7.8%
Ellsworth 2018	27	38	29	38	0.93	[0.71; 1.22]	2.0%	7.2%
Udland 2019	148	247	151	247	0.98	[0.85; 1.13]	10.3%	8.7%
Chandrasekharan 2017	56	93	328	930	1.71	[1.42; 2.06]	4.1%	8.2%
Venkata 2024	187	225	1630	3555	1.81	[1.69; 1.94]	13.3%	9.2%
**Common effect model**		**1404**		**5649**	**1.20**	**[1.15; 1.27]**	**63.2%**	**.**
**Random effects model**					**1.16**	**[0.93; 1.45]**	**.**	**58.8%**
Heterogeneity: I^2 = 96.4%, τ^2 = 0.0802, p < 0.0001								
**Common effect model**		**2500**		**6749**	**1.10**	**[1.05; 1.15]**	**100.0%**	**.**
**Random effects model**					**1.04**	**[0.90; 1.21]**	**.**	**100.0%**

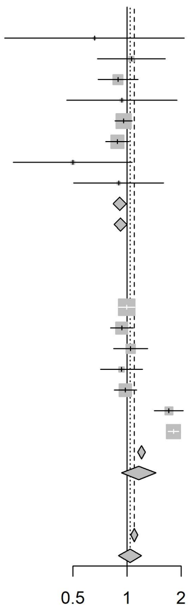

Heterogeneity: I^2 = 94.0%, τ^2 = 0.0614, p < 0.0001
Test for subgroup differences (common effect): χ^2_1 = 30.76, df = 1 (p < 0.0001)
Test for subgroup differences (random effects): χ^2_1 = 3.77, df = 1 (p = 0.0522)

(4) Death or BPD

Study	Experimental Events	Experimental Total	Control Events	Control Total	RR	95%-CI	Weight (common)	Weight (random)
**RCT**								
Meurs 2005	167	210	170	208	0.97	[0.89; 1.07]	24.6%	35.2%
Kinsella 2014	25	59	26	65	1.06	[0.70; 1.61]	3.6%	1.8%
Kinsella 2006	282	394	295	392	0.95	[0.87; 1.03]	42.6%	43.8%
Ballard 2006	165	294	183	288	0.88	[0.77; 1.01]	26.7%	17.4%
Mirza 2025	10	16	11	16	0.91	[0.55; 1.50]	1.6%	1.2%
**Common effect model**		**973**		**969**	**0.94**	**[0.89; 1.00]**	**99.1%**	**.**
**Random effects model**					**0.95**	**[0.90; 1.00]**	**.**	**99.4%**
Heterogeneity: I^2 = 0%, τ^2 = 0, p = 0.7961								
**no-RCT**								
Valerie 2009	3	6	6	6	0.54	[0.26; 1.10]	0.9%	0.6%
**Common effect model**		**979**		**975**	**0.94**	**[0.88; 0.99]**	**100.0%**	**.**
**Random effects model**					**0.94**	**[0.89; 1.00]**	**.**	**100.0%**

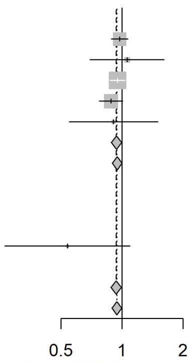

Heterogeneity: I^2 = 0.0%, τ^2 < 0.0001, p = 0.5381
Test for subgroup differences (common effect): χ^2_1 = 2.02, df = 1 (p = 0.1555)
Test for subgroup differences (random effects): χ^2_1 = 2.41, df = 1 (p = 0.1207)

(B) Subgroup analysis by specific diseases

(1) Death before discharge

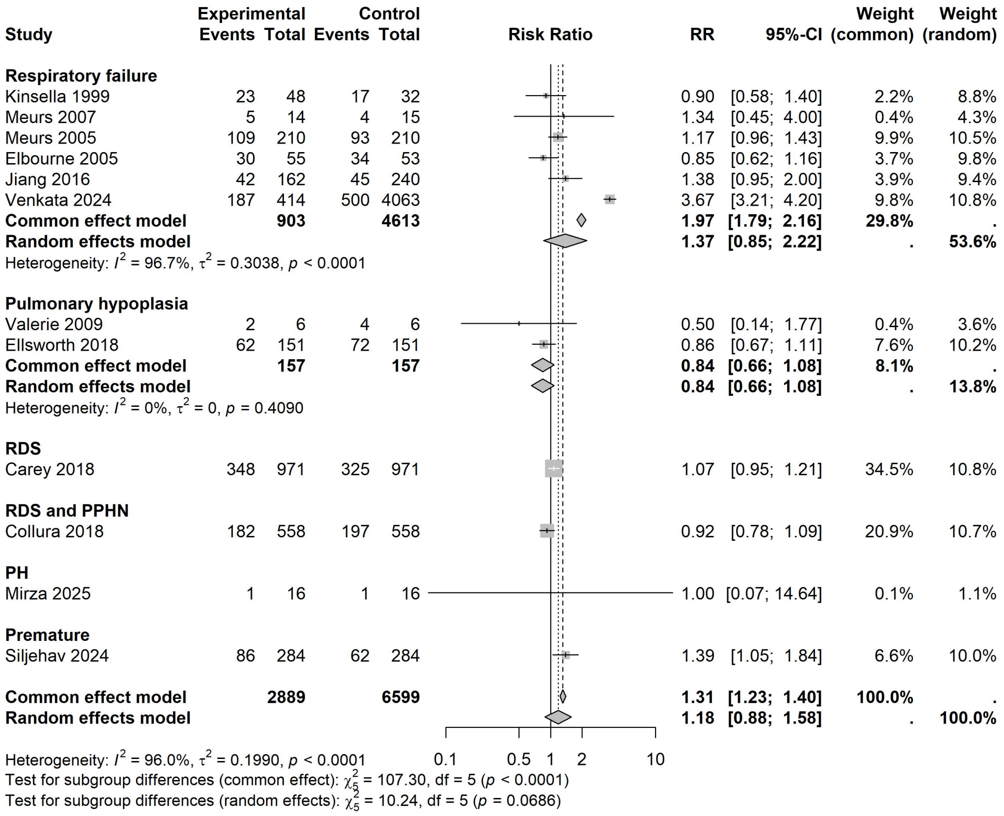

(2) Death at 36 weeks' PMA

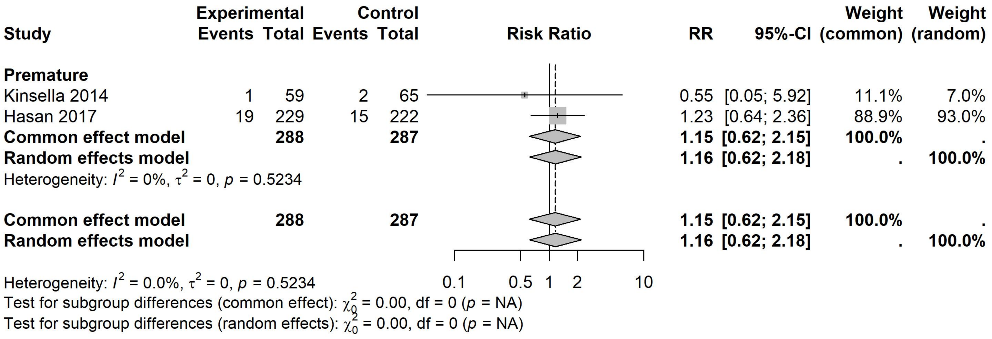

(3) BPD

Study	Experimental Events	Experimental Total	Control Events	Control Total	RR	95%-CI	Weight (common)	Weight (random)
**Respiratory failure**								
Meurs 2007	3	10	5	11	0.66	[0.21; 2.08]	0.3%	1.4%
Mercier 2010	81	339	96	358	0.89	[0.69; 1.15]	6.4%	7.4%
Su 2008	10	32	11	33	0.94	[0.46; 1.90]	0.7%	3.0%
Kinsella 2006	212	326	210	309	0.96	[0.86; 1.07]	14.8%	9.0%
Jiang 2016	75	162	106	240	1.05	[0.84; 1.30]	5.8%	7.8%
Venkata 2024	187	225	1630	3555	1.81	[1.69; 1.94]	13.3%	9.2%
**Common effect model**		**1094**		**4506**	**1.23**	**[1.16; 1.31]**	**41.3%**	**.**
**Random effects model**					**1.09**	**[0.82; 1.45]**	**.**	**37.8%**
Heterogeneity: $I^2 = 95.8\%$, $\tau^2 = 0.0903$, $p < 0.0001$								
**RDS**								
Carey 2018	263	419	264	419	1.00	[0.90; 1.11]	18.1%	9.0%
Dani 2006	6	20	12	20	0.50	[0.23; 1.07]	0.8%	2.7%
**Common effect model**		**439**		**439**	**0.97**	**[0.88; 1.08]**	**18.9%**	**.**
**Random effects model**					**0.79**	**[0.41; 1.49]**	**.**	**11.7%**
Heterogeneity: $I^2 = 67.8\%$, $\tau^2 = 0.1612$, $p = 0.0778$								
**Premature**								
Kinsella 2014	24	59	25	65	1.06	[0.68; 1.63]	1.6%	5.2%
Ballard 2006	149	294	165	288	0.88	[0.76; 1.03]	11.4%	8.6%
**Common effect model**		**353**		**353**	**0.91**	**[0.79; 1.05]**	**13.0%**	**.**
**Random effects model**					**0.90**	**[0.78; 1.04]**	**.**	**13.8%**
Heterogeneity: $I^2 = 0\%$, $\tau^2 = 0$, $p = 0.4472$								
**RDS and PH**								
Udland 2019	148	247	151	247	0.98	[0.85; 1.13]	10.3%	8.7%
**RDS and PPHN**								
Collura 2018	132	220	141	220	0.94	[0.81; 1.08]	9.6%	8.6%
**Respiratory failure /PH**								
Chandrasekharan 2017	56	93	328	930	1.71	[1.42; 2.06]	4.1%	8.2%
**Pulmonary hypoplasia**								
Ellsworth 2018	27	38	29	38	0.93	[0.71; 1.22]	2.0%	7.2%
**PH**								
Mirza 2025	9	16	10	16	0.90	[0.51; 1.60]	0.7%	3.9%
**Common effect model**		**2500**		**6749**	**1.10**	**[1.05; 1.15]**	**100.0%**	**.**
**Random effects model**					**1.04**	**[0.90; 1.21]**	**.**	**100.0%**

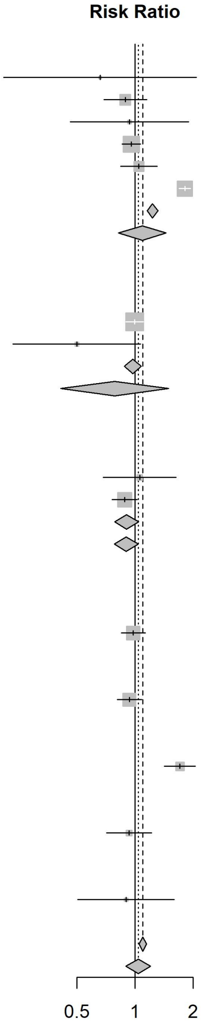

Heterogeneity: $I^2 = 94.0\%$, $\tau^2 = 0.0614$, $p < 0.0001$
Test for subgroup differences (common effect): $\chi^2_7 = 54.85$, df = 7 ($p < 0.0001$)
Test for subgroup differences (random effects): $\chi^2_7 = 35.18$, df = 7 ($p < 0.0001$)

(4) Death or BPD

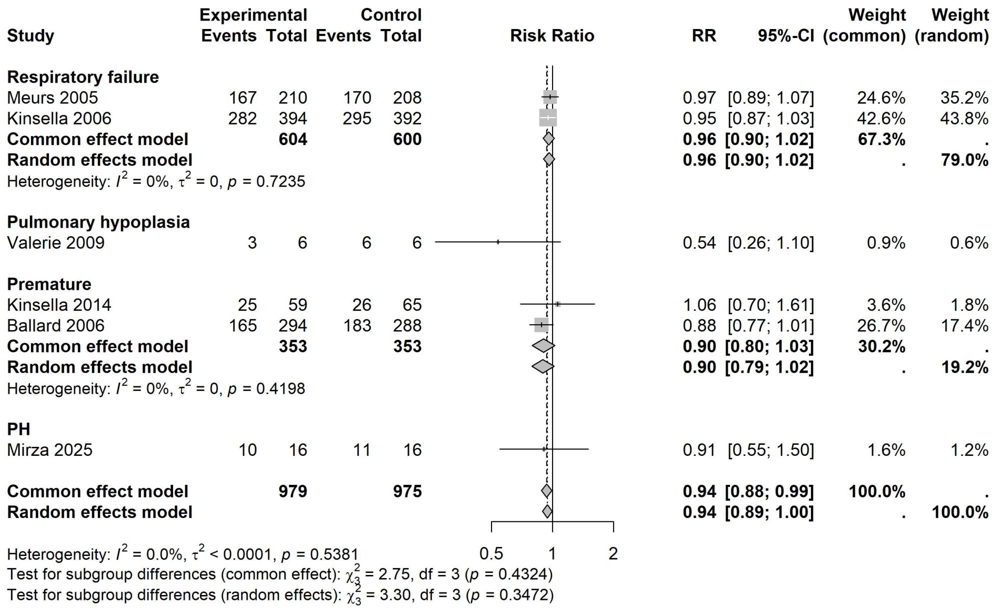

(C) Subgroup analysis by dose of iNO

(1) Change in OI after 30 min

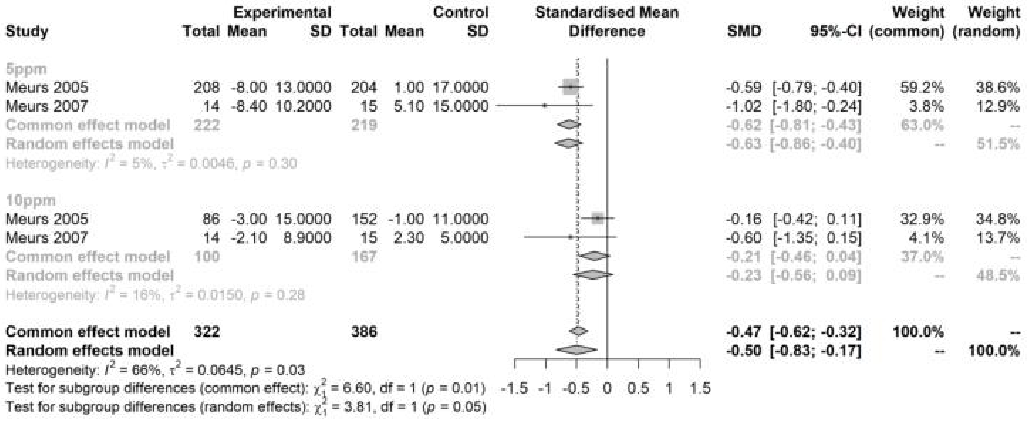

(2) Change in PaO2 after 30 min

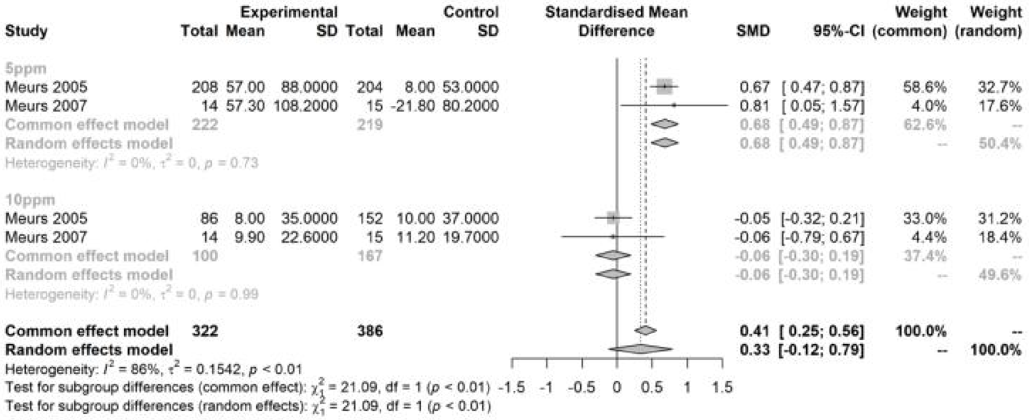

(3) Increase larger than 20 mmHg in PaO2 after 30 min

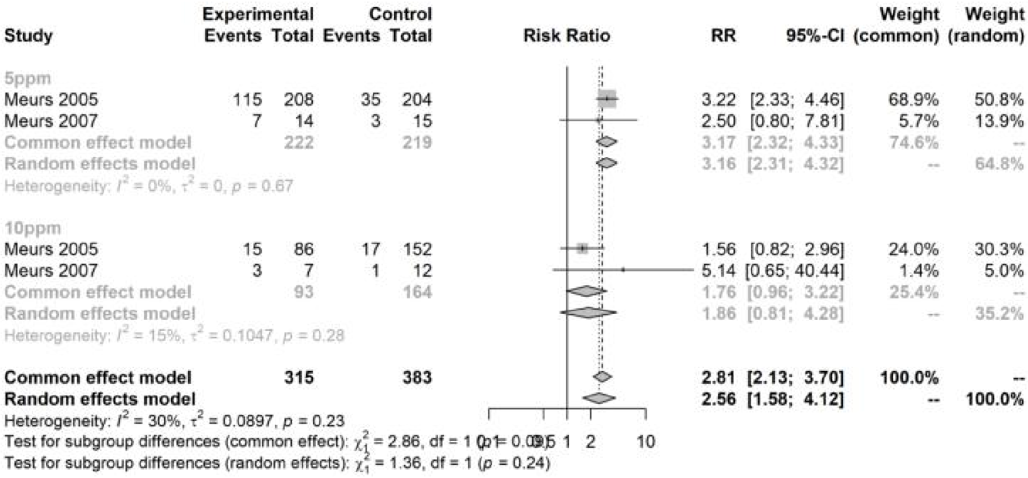

(4) Increase 10-20 mmHg in PaO2 after 30 min

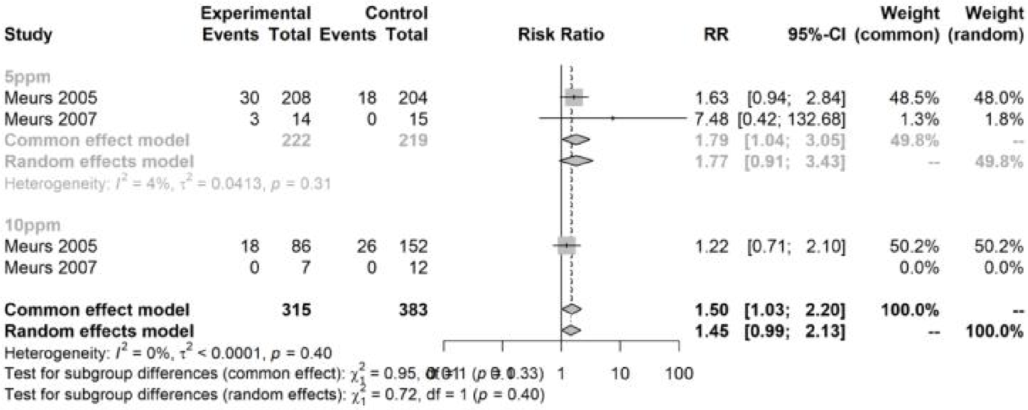

(5) Increase less than 10 mmHg in PaO2 after 30 min

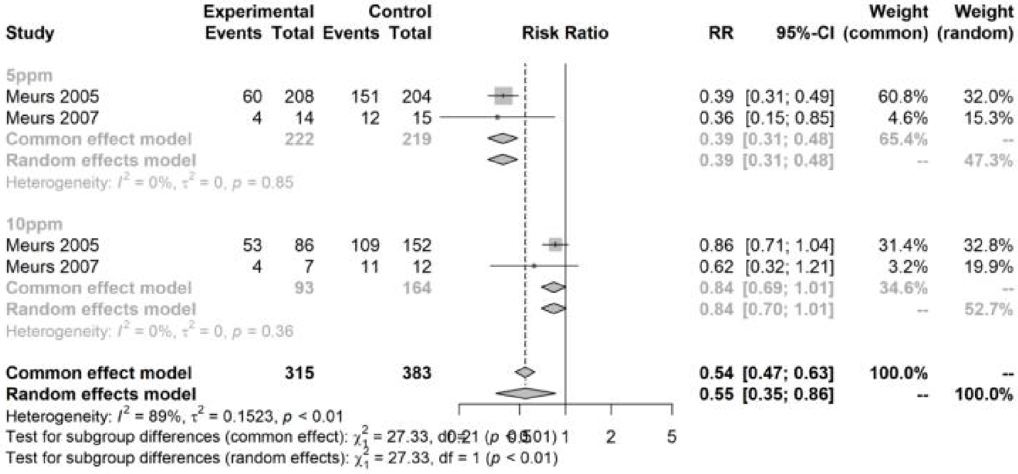

Supplement: Supplementary file 6 — Supplementary Material 6. [file 40001_2025_3008_MOESM6_ESM.pdf]
