# Supplementary material for: Inhaled nitric oxide in preterm infants with respiratory disease: a systematic review and meta-analysis
Source: Eur J Med Res. 2025 Aug 29;30:821. doi: 10.1186/s40001-025-03008-1 (PMC12395824; doi:10.1186/s40001-025-03008-1)

Appendix. File 3. Forest flots of secondary outcomes

Article title: Inhaled nitric oxide in preterm infants with respiratory disease: a systematic review and meta-analysis

Journal name: European Journal of Medical Research.

Author names: Kai Zhou, Weipeng Xu,Danrui Li, CheokUn Lao, Shiqian Zou, Shixian Liu, Bingxiao Li, Fangfang Zeng, Sui Zhu, Shasha Han.

Affiliation and e-mail address of the corresponding author: Department of Neonatology and Pediatrics, The First Affiliated Hospital of Jinan University, Guangzhou, Guangdong, China;hanssha888@163.com.

(A) Respond after 30 minutes with NO

(1) Change in OI after 30 min

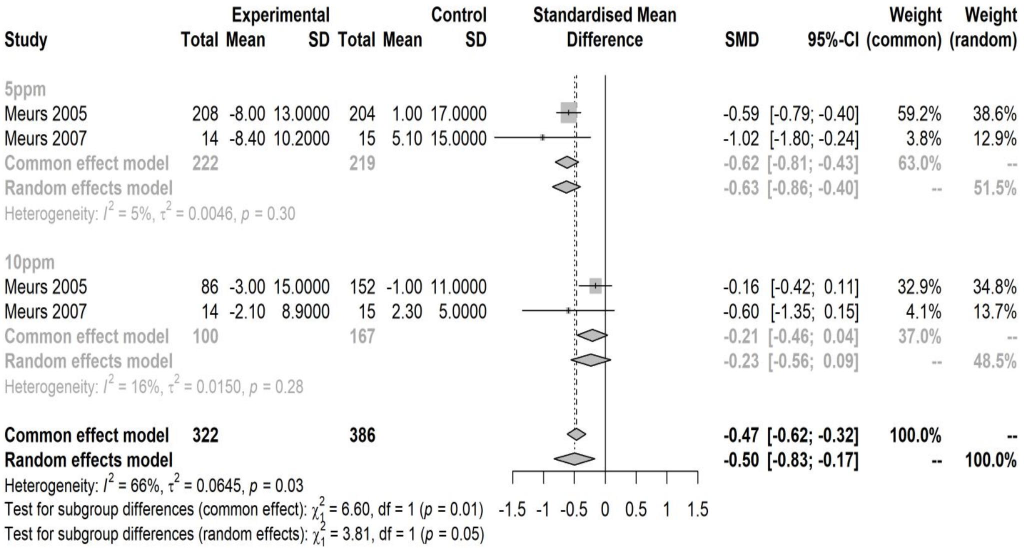

(2) Change in PaO2 after 30 min

Study	Experimental Total	Experimental Mean	Experimental SD	Control Total	Control Mean	Control SD	SMD	95%-CI	Weight (common)	Weight (random)
**5ppm**										
Meurs 2005	208	57.00	88.0000	204	8.00	53.0000	0.67	[0.47; 0.87]	58.6%	32.7%
Meurs 2007	14	57.30	108.2000	15	-21.80	80.2000	0.81	[0.05; 1.57]	4.0%	17.6%
Common effect model	222			219			0.68	[0.49; 0.87]	62.6%	--
Random effects model							0.68	[0.49; 0.87]	--	50.4%
Heterogeneity: $I^2 = 0\%$, $\tau^2 = 0$, $p = 0.73$										
**10ppm**										
Meurs 2005	86	8.00	35.0000	152	10.00	37.0000	-0.05	[-0.32; 0.21]	33.0%	31.2%
Meurs 2007	14	9.90	22.6000	15	11.20	19.7000	-0.06	[-0.79; 0.67]	4.4%	18.4%
Common effect model	100			167			-0.06	[-0.30; 0.19]	37.4%	--
Random effects model							-0.06	[-0.30; 0.19]	--	49.6%
Heterogeneity: $I^2 = 0\%$, $\tau^2 = 0$, $p = 0.99$										
**Common effect model**	**322**			**386**			**0.41**	**[0.25; 0.56]**	**100.0%**	**--**
**Random effects model**							**0.33**	**[-0.12; 0.79]**	**--**	**100.0%**

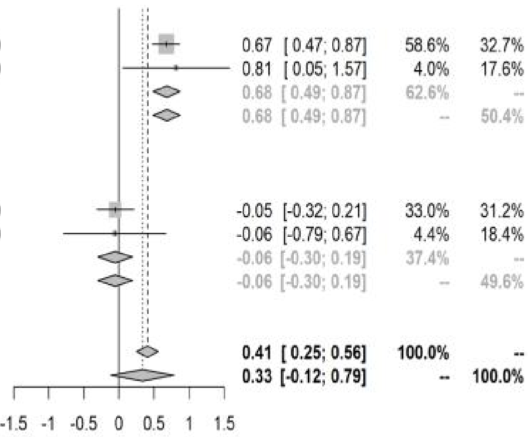

Heterogeneity: $I^2 = 86\%$, $\tau^2 = 0.1542$, $p < 0.01$
Test for subgroup differences (common effect): $\chi^2_1 = 21.09$, df = 1 ($p < 0.01$)
Test for subgroup differences (random effects): $\chi^2_1 = 21.09$, df = 1 ($p < 0.01$)

(3) Increase larger than 20 mmHg in PaO2 after 30 min

Study	Experimental Events	Experimental Total	Control Events	Control Total	RR	95%-CI	Weight (common)	Weight (random)
**5ppm**								
Meurs 2005	115	208	35	204	3.22	[2.33; 4.46]	68.9%	50.8%
Meurs 2007	7	14	3	15	2.50	[0.80; 7.81]	5.7%	13.9%
Common effect model		222		219	3.17	[2.32; 4.33]	74.6%	--
Random effects model					3.16	[2.31; 4.32]	--	64.8%
Heterogeneity: $I^2 = 0\%$, $\tau^2 = 0$, $p = 0.67$								
**10ppm**								
Meurs 2005	15	86	17	152	1.56	[0.82; 2.96]	24.0%	30.3%
Meurs 2007	3	7	1	12	5.14	[0.65; 40.44]	1.4%	5.0%
Common effect model		93		164	1.76	[0.96; 3.22]	25.4%	--
Random effects model					1.86	[0.81; 4.28]	--	35.2%
Heterogeneity: $I^2 = 15\%$, $\tau^2 = 0.1047$, $p = 0.28$								
**Common effect model**		**315**		**383**	**2.81**	**[2.13; 3.70]**	**100.0%**	**--**
**Random effects model**					**2.56**	**[1.58; 4.12]**	**--**	**100.0%**

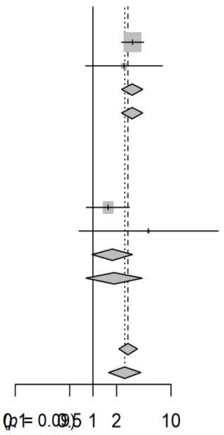

Heterogeneity: $I^2 = 30\%$, $\tau^2 = 0.0897$, $p = 0.23$
Test for subgroup differences (common effect): $\chi^2_1 = 2.86$, df = 1 ($p = 0.09$)
Test for subgroup differences (random effects): $\chi^2_1 = 1.36$, df = 1 ($p = 0.24$)

(4) Increase 10-20 mmHg in PaO2 after 30 min

Study	Experimental Events	Experimental Total	Control Events	Control Total	RR	95%-CI	Weight (common)	Weight (random)
**5ppm**								
Meurs 2005	30	208	18	204	1.63	[0.94; 2.84]	48.5%	48.0%
Meurs 2007	3	14	0	15	7.48	[0.42; 132.68]	1.3%	1.8%
Common effect model		222		219	1.79	[1.04; 3.05]	49.8%	--
Random effects model					1.77	[0.91; 3.43]	--	49.8%
Heterogeneity: $I^2 = 4\%$, $\tau^2 = 0.0413$, $p = 0.31$								
**10ppm**								
Meurs 2005	18	86	26	152	1.22	[0.71; 2.10]	50.2%	50.2%
Meurs 2007	0	7	0	12			0.0%	0.0%
**Common effect model**		**315**		**383**	**1.50**	**[1.03; 2.20]**	**100.0%**	**--**
**Random effects model**					**1.45**	**[0.99; 2.13]**	**--**	**100.0%**

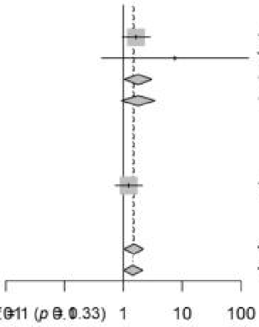

Heterogeneity: $I^2 = 0\%$, $\tau^2 < 0.0001$, $p = 0.40$
Test for subgroup differences (common effect): $\chi^2_1 = 0.95$, df = 1 ($p = 0.33$)
Test for subgroup differences (random effects): $\chi^2_1 = 0.72$, df = 1 ($p = 0.40$)

(5) Increase less than 10 mmHg in PaO2 after 30 min

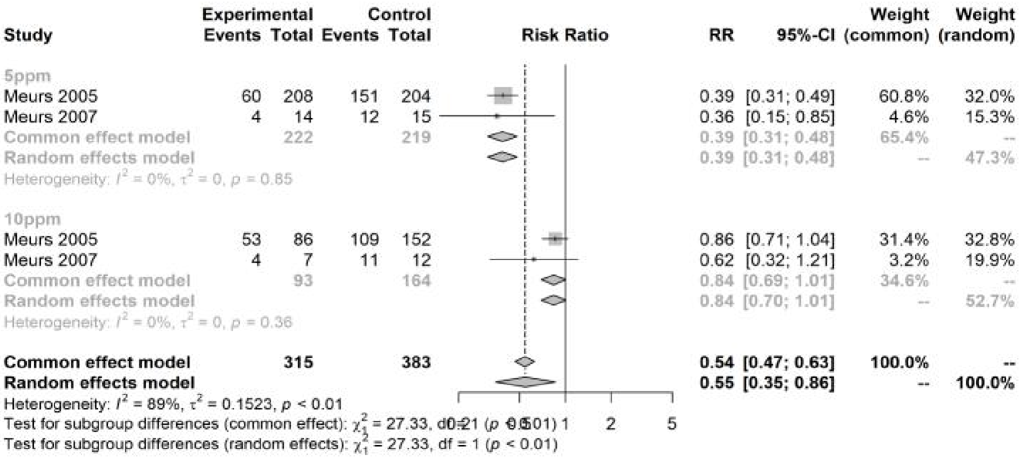

Study
Experimental Events Total
Control Events Total
Risk Ratio
RR
95%-CI
Weight (common)
Weight (random)
5ppm
Meurs 2005 60 208 151 204 0.39 [0.31; 0.49] 60.8% 32.0%
Meurs 2007 4 14 12 15 0.36 [0.15; 0.85] 4.6% 15.3%
Common effect model 222 219 0.39 [0.31; 0.48] 65.4% --
Random effects model 0.39 [0.31; 0.48] -- 47.3%
Heterogeneity: I^2 = 0%, τ^2 = 0, p = 0.85
10ppm
Meurs 2005 53 86 109 152 0.86 [0.71; 1.04] 31.4% 32.8%
Meurs 2007 4 7 11 12 0.62 [0.32; 1.21] 3.2% 19.9%
Common effect model 93 164 0.84 [0.69; 1.01] 34.6% --
Random effects model 0.84 [0.70; 1.01] -- 52.7%
Heterogeneity: I^2 = 0%, τ^2 = 0, p = 0.36
Common effect model 315 383 0.54 [0.47; 0.63] 100.0% --
Random effects model 0.55 [0.35; 0.86] -- 100.0%
Heterogeneity: I^2 = 89%, τ^2 = 0.1523, p < 0.01
Test for subgroup differences (common effect): χ^2_1 = 27.33, df = 1 (p < 0.01)
Test for subgroup differences (random effects): χ^2_1 = 27.33, df = 1 (p < 0.01)
0.2 0.5 1 2 5

(B) Respiratory system

(1) Supplemental oxygen at 1 year corrected age

Study	Experimental Events	Experimental Total	Control Events	Control Total	RR	95%-CI	Weight (common)	Weight (random)
Elbourne 2005	3	55	1	53	2.89	[0.31; 26.93]	0.7%	0.7%
Hibbs 2008	88	230	111	225	0.78	[0.63; 0.96]	75.1%	82.5%
Durrmeyer 2015	28	341	37	355	0.79	[0.49; 1.26]	24.3%	16.8%
**Common effect model**		**626**		**633**	**0.79**	**[0.65; 0.96]**	**100.0%**	--
**Random effects model**					**0.79**	**[0.65; 0.95]**	--	**100.0%**

Heterogeneity: $I^2 = 0\%$, $\tau^2 < 0.0001$, $p = 0.52$

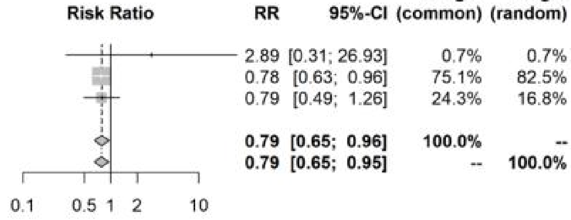

(2) Pulmonary hemorrhage

Study	Experimental Events	Experimental Total	Control Events	Control Total	RR	95%-CI	Weight (common)	Weight (random)
Kinsella 1999	6	48	3	32	1.33	[0.36; 4.95]	4.6%	5.7%
Schreiber 2003	4	105	7	102	0.56	[0.17; 1.84]	9.0%	6.8%
Elbourne 2005	4	55	5	53	0.77	[0.22; 2.72]	6.5%	6.1%
Kinsella 2006	24	398	26	395	0.92	[0.54; 1.57]	33.1%	29.2%
Su 2008	3	32	2	33	1.55	[0.28; 8.65]	2.5%	3.3%
Mercier 2010	12	395	14	397	0.86	[0.40; 1.84]	17.7%	16.0%
Jiang 2016	27	162	26	240	1.54	[0.93; 2.54]	26.6%	32.9%
**Common effect model**		**1195**		**1252**	**1.06**	**[0.79; 1.43]**	**100.0%**	--
**Random effects model**					**1.07**	**[0.78; 1.47]**	--	**100.0%**

Heterogeneity: $I^2 = 0\%$, $\tau^2 = 0.0148$, $p = 0.63$

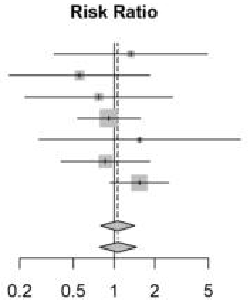

(3) Air leak

Study	Experimental Events	Experimental Total	Control Events	Control Total	RR	95%-CI	Weight (common)	Weight (random)
Schreiber 2003	39	105	54	102	0.70	[0.52; 0.96]	39.4%	39.3%
Uga 2004	2	8	1	10	2.50	[0.27; 22.86]	0.6%	1.1%
Elbourne 2005	20	55	20	53	0.96	[0.59; 1.58]	14.6%	18.7%
Kinsella 2006	25	398	24	395	1.03	[0.60; 1.78]	17.3%	15.7%
Su 2008	2	32	2	33	1.03	[0.15; 6.89]	1.4%	1.4%
Mercier 2010	12	395	13	397	0.93	[0.43; 2.01]	9.3%	8.2%
Jiang 2016	11	162	11	240	1.48	[0.66; 3.34]	6.4%	7.5%
Hasan 2017	10	229	15	222	0.65	[0.30; 1.41]	10.9%	8.1%
**Common effect model**		**1384**		**1452**	**0.88**	**[0.71; 1.08]**	**100.0%**	--
**Random effects model**					**0.87**	**[0.69; 1.09]**	--	**100.0%**

Heterogeneity: $I^2 = 0\%$, $\tau^2 = 0.0097$, $p = 0.60$

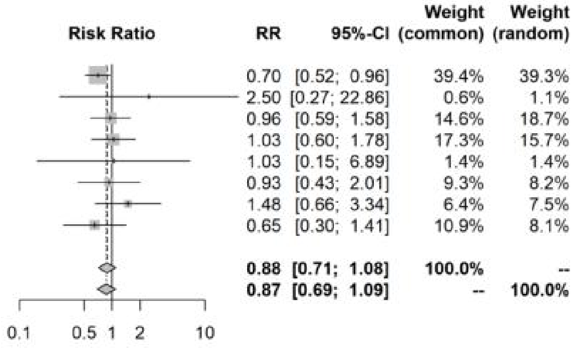

(C) Neurological developmental short-term outcomes

(1) IVH

Study	Experimental Events	Experimental Total	Control Events	Control Total	RR	95%-CI	Weight (common)	Weight (random)
Hamon 2005	7	37	5	39	1.48	[0.51; 4.24]	2.6%	2.1%
Hascoet 2005	4	61	6	84	0.92	[0.27; 3.11]	2.6%	1.6%
Dani 2006	5	20	4	20	1.25	[0.39; 3.99]	2.1%	1.7%
Mercier 2010	73	262	66	258	1.09	[0.82; 1.45]	34.9%	28.6%
Jiang 2016	84	162	124	240	1.00	[0.83; 1.22]	52.5%	62.8%
Hasan 2017	11	229	10	222	1.07	[0.46; 2.46]	5.3%	3.3%
**Common effect model**		**771**		**863**	**1.05**	**[0.90; 1.23]**	**100.0%**	--
**Random effects model**					**1.04**	**[0.89; 1.21]**	--	**100.0%**

Heterogeneity: $I^2 = 0\%$, $\tau^2 = 0$, $p = 0.98$

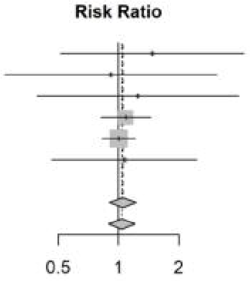

(2) IVH (GRADE 3-4)

Study	Experimental Events	Experimental Total	Control Events	Control Total	RR	95%-CI	Weight (common)	Weight (random)
Dani 2006	2	20	2	20	1.00	[0.16; 6.42]	8.3%	6.6%
Jiang 2016	26	162	25	240	1.54	[0.92; 2.57]	83.4%	86.7%
Mirza 2025	2	16	2	16	1.00	[0.16; 6.25]	8.3%	6.7%
**Common effect model**		**198**		**276**	**1.45**	**[0.90; 2.33]**	**100.0%**	.
**Random effects model**					**1.45**	**[0.90; 2.34]**	.	**100.0%**

Heterogeneity: $I^2 = 0.0\%$, $\tau^2 = 0$, $p = 0.8331$

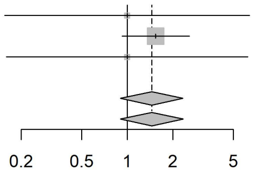

(3) PVL

Study	Experimental Events	Experimental Total	Control Events	Control Total	RR	95%-CI	Weight (common)	Weight (random)
Kinsella 2006	19	365	32	356	0.58	[0.33; 1.00]	15.2%	12.2%
Dani 2006	0	20	1	20	0.33	[0.01; 7.71]	0.7%	0.4%
Su 2008	3	32	4	33	0.77	[0.19; 3.19]	1.8%	2.0%
Mercier 2010	7	295	1	397	9.42	[1.17; 76.15]	0.4%	0.9%
Jiang 2016	12	162	27	240	0.66	[0.34; 1.26]	10.2%	8.9%
Carey 2018	73	774	67	774	1.09	[0.79; 1.49]	31.4%	31.1%
Collura 2018	43	424	37	424	1.16	[0.76; 1.77]	17.3%	19.7%
Ellsworth 2018	5	80	4	80	1.25	[0.35; 4.49]	1.9%	2.4%
Udland 2019	46	464	45	464	1.02	[0.69; 1.51]	21.1%	22.2%
**Common effect model**		**2616**		**2788**	**0.99**	**[0.83; 1.19]**	**100.0%**	--
**Random effects model**					**0.97**	**[0.80; 1.19]**	--	**100.0%**

Heterogeneity: $I^2 = 29\%$, $\tau^2 = 0.0079$, $p = 0.19$

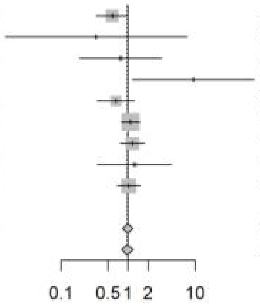

(4) Grade 3 or 4 IVH or PVL

Study	Experimental Events	Experimental Total	Control Events	Control Total	RR	95%-CI	Weight (common)	Weight (random)
Meurs 2005	69	179	50	155	1.19	[0.89; 1.60]	58.0%	64.3%
Meurs 2007	0	9	2	9	0.20	[0.01; 3.64]	2.7%	0.7%
Jiang 2016	34	162	45	240	1.12	[0.75; 1.67]	39.3%	35.0%
**Common effect model**		**350**		**404**	**1.14**	**[0.90; 1.44]**	**100.0%**	--
**Random effects model**					**1.15**	**[0.91; 1.46]**	--	**100.0%**

Heterogeneity: $I^2 = 0\%$, $\tau^2 < 0.0001$, $p = 0.48$

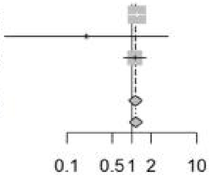

(5) ROP

Study	Experimental Events	Experimental Total	Control Events	Control Total	RR	95%-CI	Weight (common)	Weight (random)
Kinsella 1999	1	48	3	32	0.22	[0.02; 2.04]	0.5%	0.1%
Schreiber 2003	6	105	10	102	0.58	[0.22; 1.54]	1.4%	0.7%
Meurs 2005	29	98	36	112	0.92	[0.61; 1.38]	4.5%	3.6%
Elbourne 2005	8	55	4	53	1.93	[0.62; 6.02]	0.6%	0.5%
Kinsella 2006	66	398	60	395	1.09	[0.79; 1.50]	8.1%	5.6%
Ballard 2006	246	294	236	288	1.02	[0.95; 1.10]	32.2%	43.3%
Dani 2006	3	20	3	20	1.00	[0.23; 4.37]	0.4%	0.3%
Su 2008	8	32	9	33	0.92	[0.40; 2.08]	1.2%	0.9%
Kinsella 2014	3	59	4	65	0.83	[0.19; 3.54]	0.5%	0.3%
Jiang 2016	55	162	64	240	1.27	[0.94; 1.72]	7.0%	6.3%
Hasan 2017	103	187	98	184	1.03	[0.86; 1.25]	13.3%	14.2%
Chandrasekharan 2017	16	93	70	930	2.29	[1.39; 3.77]	1.7%	2.4%
Carey 2018	68	497	60	497	1.13	[0.82; 1.57]	8.1%	5.5%
Collura 2018	29	275	30	275	0.97	[0.60; 1.57]	4.1%	2.6%
Ellsworth 2018	0	44	0	44			0.0%	0.0%
Udland 2019	35	297	37	297	0.95	[0.61; 1.46]	5.0%	3.2%
Chandrasekharan 2020	63	178	235	811	1.22	[0.97; 1.53]	11.4%	10.5%
**Common effect model**		**2842**		**4378**	**1.08**	**[1.01; 1.16]**	**100.0%**	--
**Random effects model**					**1.08**	**[0.99; 1.16]**	--	**100.0%**

Heterogeneity: $I^2 = 20\%$, $\tau^2 = 0.0024$, $p = 0.22$

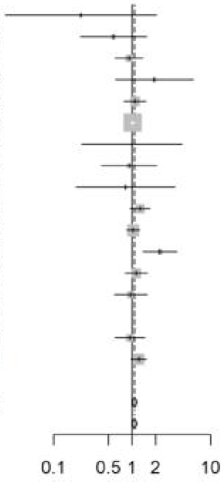

(6) ROP requiring treatment

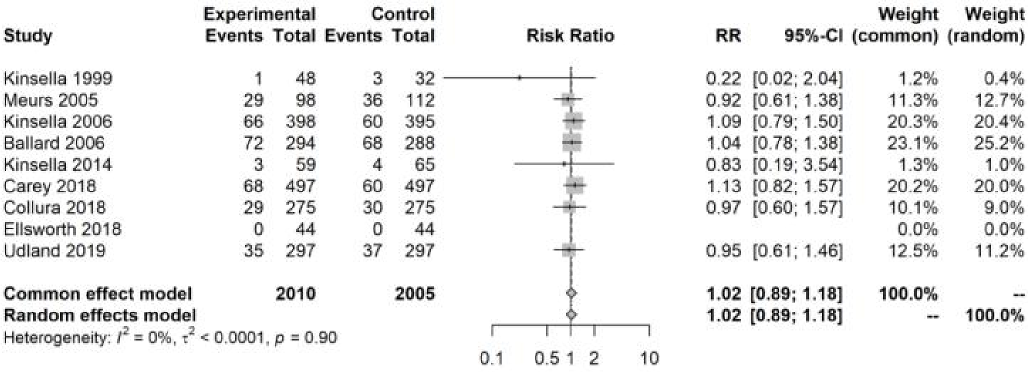

(7) Stage III and above ROP

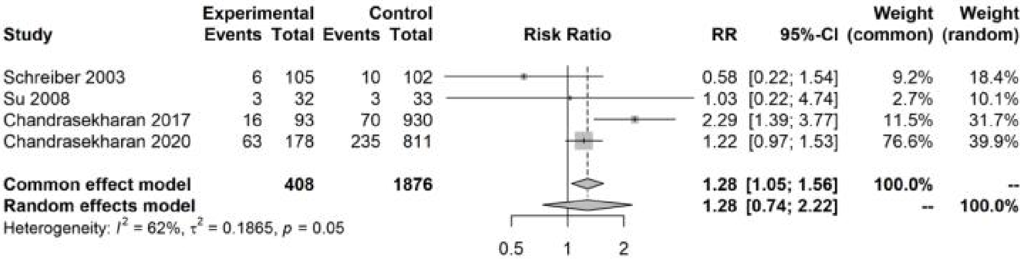

(D) Neurological developmental long-term outcomes

(1) Head circumference at 1 year corrected age

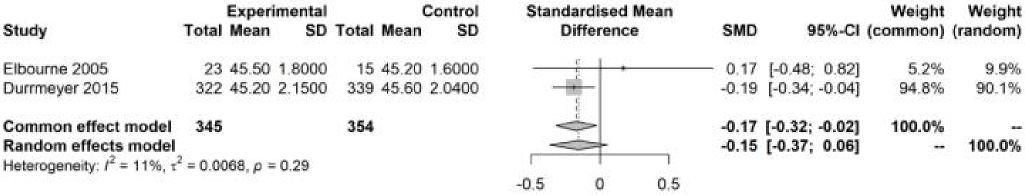

(2) CP at 18 to 26 months corrected age

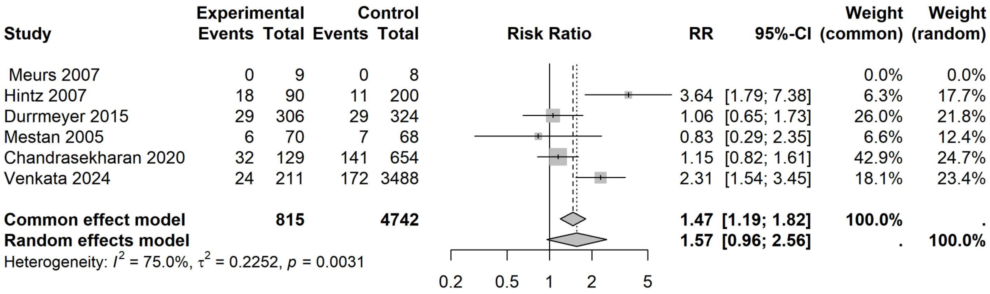

(3) NDI at 18 to 24 months corrected age

Study	Experimental Events	Experimental Total	Control Events	Control Total	RR	95%-CI	Weight (common)	Weight (random)
Meurs 2007	1	9	2	8	0.44	[0.05; 4.02]	0.6%	0.9%
Hintz 2007	45	89	48	102	1.07	[0.80; 1.44]	13.4%	24.8%
Walsh 2010	109	243	114	234	0.92	[0.76; 1.12]	34.7%	34.0%
Venkata 2024	116	213	1510	3541	1.28	[1.12; 1.45]	51.2%	40.3%
**Common effect model**		**554**		**3885**	**1.12**	**[1.01; 1.24]**	**100.0%**	**.**
**Random effects model**					**1.08**	**[0.88; 1.34]**	**.**	**100.0%**

Heterogeneity: $I^2 = 65.2\%$, $\tau^2 = 0.0239$, $p = 0.0346$

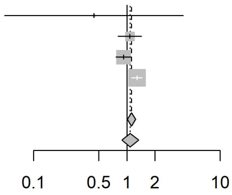

(4) Loss of vision at 18 to 26 months corrected age

Study	Experimental Events	Experimental Total	Control Events	Control Total	RR	95%-CI	Weight (common)	Weight (random)
Mestan 2005	0	70	2	68	0.19	[0.01; 3.97]	13.4%	5.0%
Meurs 2007	0	9	0	8			0.0%	0.0%
Hintz 2007	2	90	1	102	2.27	[0.21; 24.58]	5.0%	8.0%
Walsh 2010	9	243	9	234	0.96	[0.39; 2.38]	48.5%	55.5%
Chandrasekharan 2020	3	129	19	652	0.80	[0.24; 2.66]	33.2%	31.5%
**Common effect model**		**541**		**1064**	**0.87**	**[0.45; 1.68]**	**100.0%**	**--**
**Random effects model**					**0.90**	**[0.46; 1.76]**	**--**	**100.0%**

Heterogeneity: $I^2 = 0\%$, $\tau^2 = 0$, $p = 0.65$

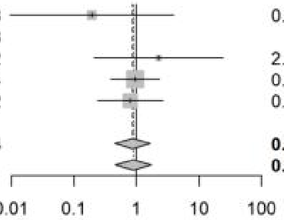

(5) Visual impairment at 18-26 months corrected age

Study	Experimental Events	Experimental Total	Control Events	Control Total	RR	95%-CI	Weight (common)	Weight (random)
Meurs 2007	0	9	0	8			0.0%	0.0%
Hintz 2007	2	90	1	102	2.27	[0.21; 24.58]	1.1%	1.1%
Durrmeyer 2015	79	306	65	324	1.29	[0.96; 1.72]	74.2%	78.3%
Mestan 2005	0	70	2	68	0.19	[0.01; 3.97]	3.0%	0.7%
Walsh 2010	9	243	9	234	0.96	[0.39; 2.38]	10.8%	7.9%
Chandrasekharan 2020	3	129	19	652	0.80	[0.24; 2.66]	7.4%	4.5%
Venkata 2024	5	196	27	3259	3.08	[1.20; 7.91]	3.6%	7.3%
**Common effect model**		**1043**		**4647**	**1.26**	**[0.98; 1.62]**	**100.0%**	**.**
**Random effects model**					**1.30**	**[1.01; 1.68]**	**.**	**100.0%**

Heterogeneity: $I^2 = 16.7\%$, $\tau^2 < 0.0001$, $p = 0.3062$

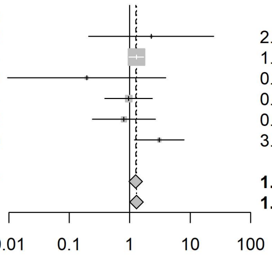

(E) Digestive system

(1) NEC

Study	Experimental Events	Experimental Total	Control Events	Control Total	RR	95%-CI	Weight (common)	Weight (random)
Schreiber 2003	13	105	6	102	2.10	[0.83; 5.32]	1.6%	2.0%
Hamon 2005	4	37	4	39	1.05	[0.28; 3.91]	1.0%	1.0%
Hascoet 2005	5	61	5	84	1.38	[0.42; 4.55]	1.1%	1.2%
Kinsella 2006	53	379	46	369	1.12	[0.78; 1.62]	12.0%	12.8%
Ballard 2006	23	294	19	288	1.19	[0.66; 2.13]	4.9%	5.0%
Dani 2006	1	20	0	20	3.00	[0.13; 69.42]	0.1%	0.2%
Su 2008	2	32	2	33	1.03	[0.15; 6.89]	0.5%	0.5%
Mercier 2010	11	397	7	395	1.56	[0.61; 3.99]	1.8%	2.0%
Kinsella 2014	5	59	10	65	0.55	[0.20; 1.52]	2.5%	1.7%
Jiang 2016	12	162	23	240	0.77	[0.40; 1.51]	4.8%	3.9%
Hasan 2017	17	229	18	222	0.92	[0.48; 1.73]	4.7%	4.3%
Chandrasekharan 2017	18	93	116	930	1.55	[0.99; 2.43]	5.4%	8.6%
Carey 2018	112	971	107	971	1.05	[0.82; 1.34]	27.6%	27.8%
Collura 2018	67	558	59	558	1.14	[0.82; 1.58]	15.2%	15.9%
Ellsworth 2018	7	151	8	151	0.88	[0.33; 2.35]	2.1%	1.8%
Chandrasekharan 2020	27	338	145	1393	0.77	[0.52; 1.14]	14.6%	11.2%
Mirza 2025	3	16	0	16	7.00	[0.39; 125.14]	0.1%	0.2%
**Common effect model**		**3902**		**5876**	**1.07**	**[0.94; 1.22]**	**100.0%**	**.**
**Random effects model**					**1.08**	**[0.94; 1.23]**	**.**	**100.0%**

Heterogeneity: $I^2 = 0.0\%$, $\tau^2 = 0$, $p = 0.6325$

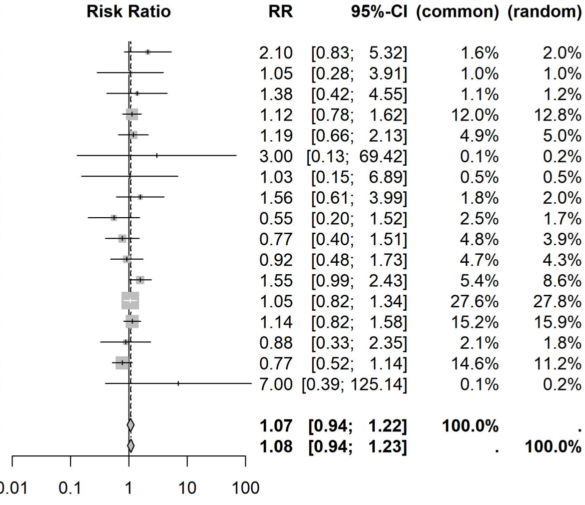

(F) Circular system

(1) Symptomatic PDA

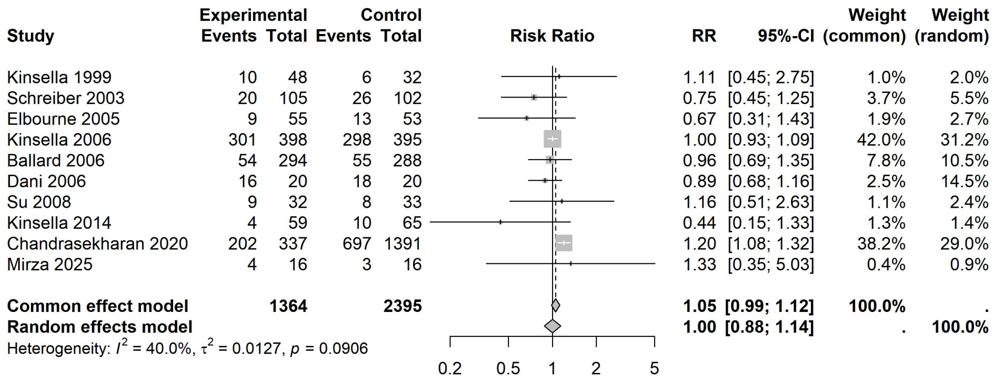

(2) Vasopressor use

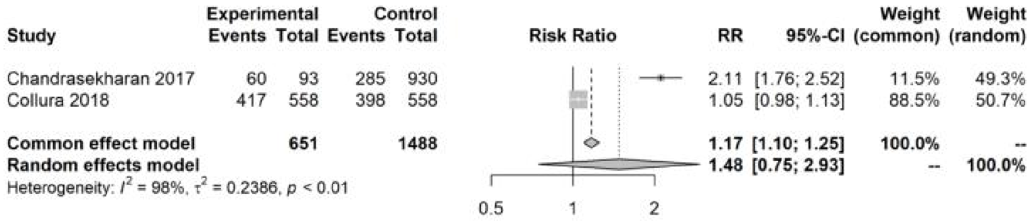

(G) Postnatal steroids

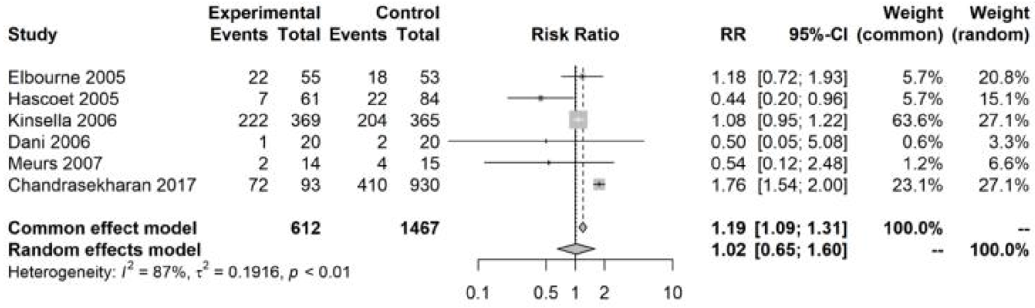

(H) Duration of hospitalization in survivors (days)

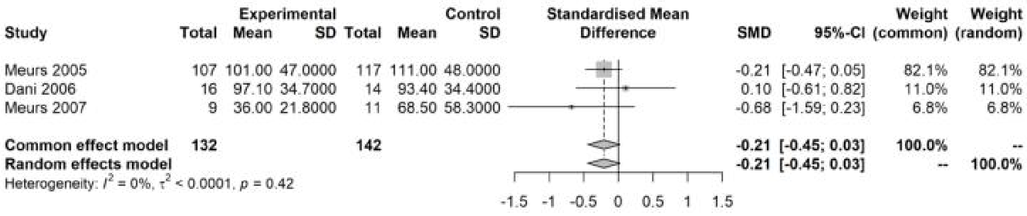

Supplement: Supplementary file 7 — Supplementary Material 7. [file 40001_2025_3008_MOESM7_ESM.pdf]
