# Supplementary material for: Inhaled nitric oxide in preterm infants with respiratory disease: a systematic review and meta-analysis
Source: Eur J Med Res. 2025 Aug 29;30:821. doi: 10.1186/s40001-025-03008-1 (PMC12395824; doi:10.1186/s40001-025-03008-1)
Supplement: Supplementary file 8 — Supplementary Material 8. [file 40001_2025_3008_MOESM8_ESM.pdf]

Appendix. File 4. Bias analysis

Article title: Inhaled nitric oxide in preterm infants with respiratory disease: a systematic review and meta-analysis

Journal name: European Journal of Medical Research.

Author names: Kai Zhou, Weipeng Xu,Danrui Li, CheokUn Lao, Shiqian Zou, Shixian Liu, Bingxiao Li, Fangfang Zeng, Sui Zhu, Shasha Han.

Affiliation and e-mail address of the corresponding author: Department of Neonatology and Pediatrics, The First Affiliated Hospital of Jinan University, Guangzhou, Guangdong, China;hanssha888@163.com.

	Random sequence generation (selection bias)	Allocation concealment (selection bias)	Blinding of participants and personnel (performance bias)	Blinding of outcome assessment (detection bias)	Incomplete outcome data (attrition bias)	Selective reporting (reporting bias)	Other bias
Ballard 2006	+	+	+		+	+	+
Dani 2006	+		+		+	+	+
Durrmeyer 2015	+		+		+	+	+
Elbourne 2005		−	−		+	+	+
Hamon 2005	+	+			+	+	+
Hasan 2017	+	+	+		+	+	+
Hascoet 2005	+		+		+	+	+
Hibbs 2008	+		+	+	+	+	+
Hintz 2007	+	+	−	+	+	+	+
Kinsella 1999	+	+	+		+	+	+
Kinsella 2006	+	+	+	+	+	+	+
Kinsella 2014	+	+			+	+	+
Mercier 2010	+	+	+	+	+	+	+
Mestan 2005	+			+	+	+	+
Meurs 2005	+	+	+	+	+	+	+
Meurs 2007	+	+	+		+	+	+
Mirza 2025			+			+	+
Schreiber 2003	+		+	+	+	+	+
Su 2008	+		−		+	+	+
Walsh 2010	+			+	+	+	+

+, low risk; −, high risk; blank, unclear risk of bias.

Risk of bias of individual studies for RCTs.

Risk of bias of individual studies for observational studies.

		Selection				Comparability	Outcome				
**Author**	**Year**	**Representativeness of the exposed cohort**	**Selection of the non- exposed cohort**	**Ascertainment of exposure**	**Demonstration that the outcome of interest was not present at the start of the study**	**Comparability of cohorts on the basis of design or analysis**	**Assessment of outcome**	**Was follow-up long enough for the outcome to occur?**	**Adequacy of follow up of cohorts**	**Total stars**	**Good Quality**
Valerie	2009	*	*	*	*	*		*	*	**7**	**Yes**
Uga	2004	*	*	*	*	*		*	*	**7**	**Yes**
Udland	2019	*	*	*	*	*		*	*	**7**	**Yes**
Carey	2018	*	*		*	*	*	*	*	**7**	**Yes**
Collura	2018	*	*		*	*	*	*	*	**7**	**Yes**
Jiang	2016	*	*	*	*	*	*	*	*	**8**	**Yes**
Chandrasekharan	2017	*	*	*	*	*	*	*	*	**8**	**Yes**
Ellsworth	2018	*	*		*	*	*	*	*	**7**	**Yes**
Chandrasekharan	2020	*	*		*	*	*	*	*	**7**	**Yes**
Venkata	2024	*	*	*	*	*		*	*	**7**	**Yes**
Siljehav	2024	*	*	*	*	*	*	*	*	**8**	**Yes**

Observational studieswere assessed against 3 key domains: population selection, the comparability of the “exposed” and “comparator” groups, and outcome assessment (for cohort studies) or exposure assessment (for case control studies). Five stars or more was considered good quality.
